# Propagating instabilities in long collapsible tubes of nonlinear biological material

**DOI:** 10.1007/s10237-025-01973-4

**Published:** 2025-06-17

**Authors:** Aris G. Stamou, Ilias Gavriilidis, Ioanna D. Karetsa, Spyros A. Karamanos

**Affiliations:** Department of Mechanical Engineering, University of Thessaly, Volos, Greece

**Keywords:** Structural instability, Buckling, Collapse, Buckle propagation, Collapsible tube, Biological tube

## Abstract

Proper functionality of human body relies on several continuous physical processes, many of which are carried out through biological ducts/tubes. For instance, veins, arteries and airways into the human body are natural conduit systems where blood and air are conveyed. Those elastic tubular components are prone to structural instability (buckling) and eventually collapse under critical conditions of net external pressure, resulting in malfunctioning of main physical processes. In the present work, collapsible elastic tubes are studied from a structural mechanics perspective, examining their resistance to collapse under uniform external pressure, emphasizing on the influence of nonlinear material behavior. The problem is approached numerically using nonlinear finite element models, to analyze tubes with diameter-to-thickness ratio ranging from 9 to 30, considering different nonlinear elastic material properties and focusing on the post-buckling phenomenon of “buckling propagation”. It is demonstrated that small softening deviations from linear elastic behavior may cause a localized collapse pattern followed by its propagation along the tube with a pressure lower than the collapse pressure. Results from two-dimensional (ring) and more rigorous three-dimensional (3*D*) finite element models are obtained in terms of the collapse pressure value and the propagation pressure value, i.e., the minimum pressure required for a localized buckling pattern to propagate, and the two models provide very similar predictions. A simple analytical model is also employed to explain the phenomenon of collapse localization and its subsequent propagation. In addition, special emphasis is given on the correlation between the 3*D* results and those from ring analysis in terms of the propagation profile and the energy required for the collapse pattern to advance. Finally, comparison with numerical results from tubes made of elastic–plastic material is performed to elucidate some special features of the propagation phenomenon.

## Introduction

**Fig. 1:**
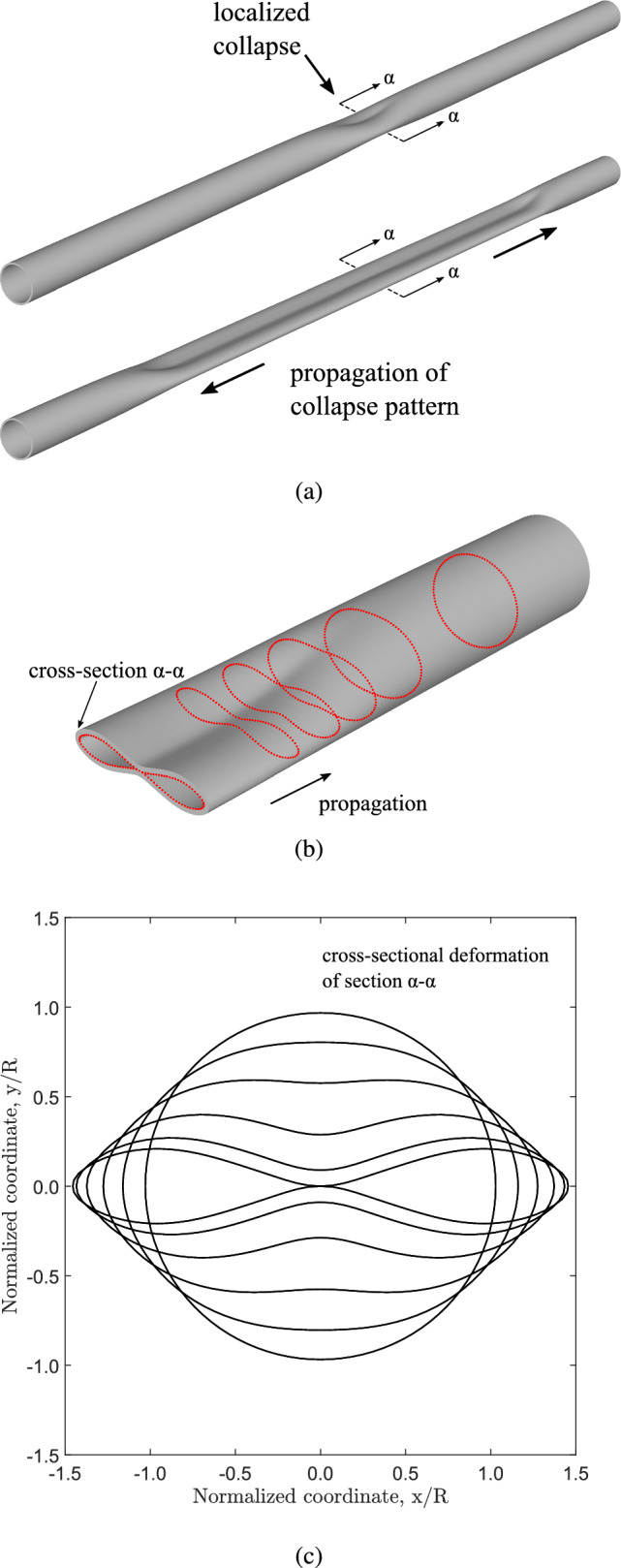
Finite element analysis of localized collapse and its propagation in a steel pipe under quasi-static steady-state conditions ($D/t=20$, *X*65 steel) under uniform external pressure **a** three-dimensional view, **b** propagation profile and consecutive stages of cross-sectional deformation, and **c** inner surface of cross section $\alpha$-$\alpha$ at different post-buckling stages; because of steady-state propagation, this also corresponds to the longitudinal view of the propagation profile (Gavriilidis et al. 2024)

The problem of collapse instability (buckling) in elongated hollow cylinders under external pressure has been studied extensively because of its special interest in the design of deep sea pipelines (Kyriakides and Corona 2007; Kyriakides and Lee 2020; Karamanos 2022). In a long metal tube, buckling may initiate in the form of a local collapse pattern at a certain cross section, as shown in Fig. 1a, and it is often referred to as “local buckle” or simply “buckle”. Buckle initiation is associated with the maximum level of external pressure that the tube is capable to sustain, and is followed by cross-sectional ovalization leading to collapse. This maximum pressure is called “buckling pressure” or “collapse pressure” $P_{{{\text{co}}}}$, and is always lower than the theoretical bifurcation or “critical” value $P_{{{\text{cr}}}}$ of a linear elastic ring, representing a slice of a long tube with perfect geometry, expressed by the following formula:

1 $$\begin{aligned} P_{{{\text{cr}}}} = \frac{2 E}{\left( 1-\nu ^{2}\right) } \left( \frac{t}{D_{{{\text{o}}}} -t}\right) ^{3} \end{aligned}$$

where $D_{{{\text{o}}}}$ is the outer diameter of the tube, *t* is tube wall thickness, *E* is Young’s modulus and $\nu$ is Poisson’s ratio (Bryan 1889). The value of collapse pressure is an essential property of the tube, representing its ultimate strength under external pressure, and depends on its geometric and material properties.

**Table 1:** Residual gap during propagation for different material nonlinearity, considering nonlinear elastic and hyperelastic material models ($D/t=20$)

Material model	*n* = 1	*n* = 2	*n* = 2.4	*n* = 2.6	*n* = 2.8
Nonlinear elastic	–	0.729	0.228	0.059	0
Hyperelastic	–	0.932	0.269	0.115	0.004

**Table 2:** Collapse and propagation pressure (in kPa) for the four *D*/*t* ring models, using the nonlinear elastic model with $n = 2.4$ and the Marlow hyperelastic material model; propagation pressure is calculated through Maxwell line construction

Material model	Collapse pressure (kPa)				Propagation pressure (kPa)			
	*D*/*t*=9	*D*/*t*=12	*D*/*t*=20	*D*/*t*=30	*D*/*t*=9	*D*/*t*=12	*D*/*t*=20	*D*/*t*=30
Nonlinear elastic	8.677	3.992	0.934	0.284	6.891	3.240	0.823	0.268
Hyperelastic	8.440	3.992	0.928	0.283	7.069	3.269	0.815	0.265

Once a local collapsed pattern is established, it has the potential to propagate along the tube length (Fig. 1a), a phenomenon known as “buckling propagation”. Buckling propagation is of major concern for the structural integrity of subsea pipelines due to its catastrophic consequences (Kyriakides and Lee 2020; Karamanos 2022). Under buckling propagation, the deformed configuration of the tube is considered as a sequence of collapsing rings along its length, as shown in Fig. 1b. A longitudinal view of the propagation profile at cross section $\alpha$-$\alpha$ is shown in Fig. 1c which also corresponds to the consecutive deformed configurations of cross section $\alpha$-$\alpha$ at different stages. A main feature of buckling propagation is that the minimum level of external pressure, necessary for the buckle to propagate, known as “propagation pressure” ($P_{{{\text{p}}}}$), is significantly lower than the collapse pressure of the tube $P_{{{\text{co}}}}$ ($P_{{{\text{p}}}} < P_{{{\text{co}}}}$). If the externally applied pressure is equal to $P_{{{\text{p}}}}$ then the propagation is quasi-static. If it is higher than the value of $P_{{{\text{p}}}}$, then dynamic propagation occurs with a speed that depends on the level of pressure.

Collapsible tubes are also encountered in biomechanical engineering, and refer to numerous biological “pipe-like” or “flow” systems in the human body. These applications involve structural stability issues associated with collapsible tubes (Heil and Pedley 1996; Heil 1997; Han et al. 2013; Kozlovsky et al. 2014); typical examples of physiological cylindrical vessels in the human body are the air flow through airways during mandated exhalation or due to obstructive sleeping apnea (Isono 2012), the blood flow through arteries and veins, and the pulmonary capillaries, the urethra during micturition and the ureter during peristaltic pumping (Kamm and Pedley 1989; Pedley 1992; Bertram and Elliott 2003; Marzo et al. 2005). Han et al. (2013) reported in detail the most common forms of buckling that occur in blood vessels and conducts, including cross-sectional collapse, longitudinal twist buckling, bent buckling, kinking and helical buckling. It has been recognized that biological cylinders conveying fluid (air or blood) are quite flexible and susceptible to structural instability in the form of collapse due to net external transmural pressure ($P= P_{{{\text{out}}}} - P_{{{\text{in}}}}$) resulted from the internal pressure ($P_{{{\text{in}}}}$) of the containment and the external pressure ($P_{{{\text{out}}}}$) of the outer environment. Upon reaching a buckling stage under external pressure ($P_{{{\text{co}}}}$), the post-buckling deformation pattern of the tube cross section is significantly ovalized and sometimes completely collapsed, when the opposite sides of internal surface of the tube establish contact. Under those circumstances, the cross-sectional area of the physiological conduct is significantly diminished, and the fluid flow through the tube is impended or becomes highly irregular (Kamm and Pedley 1989; Bertram 2004). Those flow-structure interactions may lead to flow obstructions that depend on the Reynolds number (Marzo et al. 2005; Bertram and Elliott 2003; Chowdhury and Zhang 2024), and are responsible for the initiation of self-excited, flow-induced oscillations of the tube.

**Table 3:** Collapse and propagation pressure (in kPa) for the four *D*/*t* 3*D* tube models, the nonlinear elastic model with $n=2.4$ and the Marlow hyperelastic material model

Material model	Collapse pressure (kPa)				Propagation pressure (kPa)			
	*D/t*= 9	*D/t*= 12	*D/t* = 20	*D/t* = 30	*D/t* = 9	*D/t* = 12	*D/t* = 20	*D/t* = 30
Nonlinear elastic	8.694	4.003	0.937	0.285	6.904	3.246	0.824	0.269
Hyperelastic	8.458	3.933	0.931	0.284	7.085	3.278	0.817	0.266

**Table 4:** Collapse and propagation pressure (in kPa) predictions for ring models ($D/t=12, 30$), using nonlinear elastic and hyperelastic material models

Material model	*D/t*	Collapse pressure (kPa)					Propagation pressure (kPa)				
		*n* = 1	*n* = 2	*n* = 2.4	*n* = 2.6	*n* = 2.8	*n* = 1	*n* = 2	*n* = 2.4	*n* = 2.6	*n* = 2.8
Nonlinear elastic	12	–	4.345	3.992	3.778	3.560	–	4.277	3.240	2.699	2.263
	30	–	–	0.284	0.282	0.279	–	–	0.268	0.244	0.218
Hyperelastic	12	–	4.371	3.922	3.662	3.400	–	4.347	3.269	2.707	2.250
	30	–	–	0.283	0.280	0.279	–	–	0.265	0.239	0.212

Structural instability phenomena in biological conduits under internal flow have been observed experimentally (Bertram 1987; Bertram et al. 1990), numerically (Heil and Pedley 1996; Downing and Ku 1997; Marzo et al. 2005; Zhu et al. 2008; Garcia et al. 2017) or using combined experimental-numerical approaches (Heil 1997; Kozlovsky et al. 2014). The aforementioned studies have been motivated by the need for simulating flow in collapsible tubes. In most of these relevant studies, the coupled fluid-tube problem is studied using fluid–structure interaction models that employ the one-dimensional fluid continuity and momentum equations, combined with the so-called “tube law”. The latter is the equilibrium diagram (or path) of net external pressure (*P*) in terms of the change of cross-sectional area ($\Delta A$) of a collapsing ring, characterizes the structural response of a tube under external pressure, considering that the tube is composed by a sequence of deformable rings.

The present paper focuses on the structural response of elastic tubes, i.e., on the “tube law”, which is a part of the complete fluid–structure interaction problem. The paper addresses some important features of tube collapse phenomena. Despite the rather extensive literature on collapse and buckle propagation in metal tubes, these instabilities have received much less attention in conduits made of biological materials. Limited numerical results have shown that tubes with linear elastic material collapse uniformly along their length and do not exhibit propagating buckles (Dyau and Kyriakides 1993). However, calculations in tubes made of bilinear elastic–plastic material with high hardening modulus (i.e., within 1/100 and 1/2 of Young’s modulus) have indicated that in those tubes localization and propagation of collapse patterns is possible, with a configuration different than the one observed in typical metal tubes, underlining the significant influence of material nonlinearity on tube structural response (Dyau and Kyriakides 1993).

**Table 5:** Collapse and propagation pressure (in kPa) predictions for 3*D* tube models ($D/t=12, 30$), using nonlinear elastic and hyperelastic material models

Material model	*D/t*	Collapse pressure (kPa)					Propagation pressure (kPa)				
		*n*=1	*n*=2	*n*=2.4	*n*=2.6	*n*=2.8	*n*=1	*n*=2	*n*=2.4	*n*=2.6	*n*=2.8
Nonlinear elastic	12	–	4.356	4.003	3.791	3.569	–	4.294	3.246	2.705	2.264
	30	–	–	0.285	0.283	0.280	–	–	0.269	0.245	0.219
Hyperelastic	12	–	4.381	3.933	3.674	3.410	–	4.378	3.278	2.713	2.254
	30	–	–	0.284	0.281	0.279	–	–	0.266	0.241	0.212

**Table 6:** Collapse and propagation pressure (in kPa) predictions for the ring and 3*D* tube models ($D/t=20$ and $n=2.4$) with no pre-strain ($0 \%$) and three levels of pre-strain ($1 \%$,$2 \%$ and $3 \%$)

Model	Collapse pressure (kPa)				Propagation pressure (kPa)			
	$0\%$	$1\%$	$2\%$	$3\%$	$0\%$	$1\%$	$2\%$	$3\%$
Ring	0.934	0.874	0.800	0.738	0.823	0.808	0.776	0.736
3*D* tube	0.937	0.878	0.803	0.741	0.824	0.811	0.780	0.742

**Table 7:** Normalized strain energy of the ring model ($\hat{U}_{r}/\hat{U}$) and the 3*D* tube model ($\hat{U}_{t}/\hat{U}$) for nonlinear elastic and metal material properties

Model	Nonlinear elastic $(n=2.4)$	Metal (*X*65 steel)
Ring	0.738	0.125
3*D* tube	0.733	0.197
Difference	$0.7\%$	$36.5\%$

The study presented in this paper is aimed at identifying the conditions under which local collapse and its propagation may be possible in collapsible tubes made of nonlinear elastic material, and investigates the effect of material nonlinearity on elastic tube structural response. In particular, the work focuses on the structural stability of externally pressurized tubes made of soft biological material, which are encountered very frequently in bioengineering applications. The tubes are long in the sense that their response is independent of any end conditions. The problem is tackled from a structural mechanics perspective, considering the experience of the research team on the structural response of metal tubes and pipes (Karamanos 2022). The study is numerical, based on nonlinear finite element simulations, and refers to elastic tubes with diameter-to-thickness ratio *D*/*t* between 9 and 30 subjected to external pressure. The analysis is quasi-static, aimed at determining the collapse pressure and the propagation pressure of the elastic tubes, excluding dynamic propagation phenomena.

Three-dimensional (3*D*) and two-dimensional (ring) finite element models are developed for the purposes of the present study. Nonlinear elastic material behavior is considered with small softening deviations from linear elastic behavior and its effect on both collapse and propagation is examined. In this context, two material models are employed: the $J_{2}$ deformation theory model and the Marlow hyperelastic model. Numerical results for the collapse pressure and the propagation pressure are obtained for a wide range of material and geometric parameters. A simple analytical model that explains the phenomenon of collapse localization in long tubes and its propagation is also developed and discussed. The finite element results aim at quantifying the effects of elastic material nonlinearity on collapse localization and subsequently on its propagation, and identifying the boundaries between stable and unstable post-buckling behavior. Finally, comparison with tubes made of nonelastic material is also conducted.

**Fig. 2:**
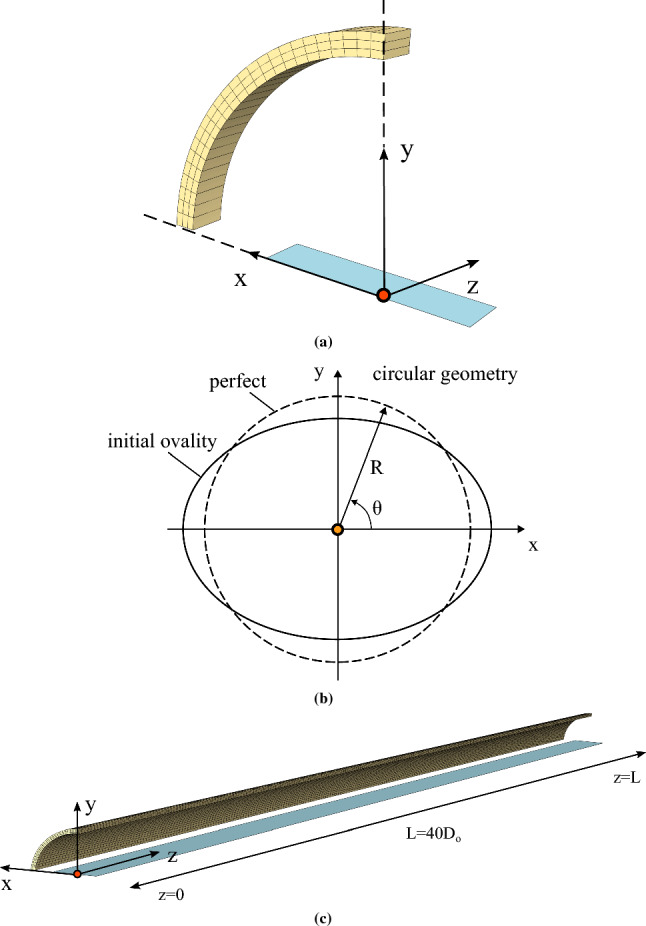
**a** Ring finite element model ($D/t=20$); one slice of a long tube is considered and one quarter is simulated due to doubly symmetric shape of cross-sectional ovalization, **b** initial cross-sectional ovalization expressed by a doubly symmetric trigonometric function of radial displacement $w_{o}$, and **c** three-dimensional (3*D*) finite element model to simulate the onset of collapse and subsequent buckling propagation ($D/t=20$); one quarter of the tube is simulated due to doubly symmetric shape of cross-sectional ovalization

**Fig. 3:**
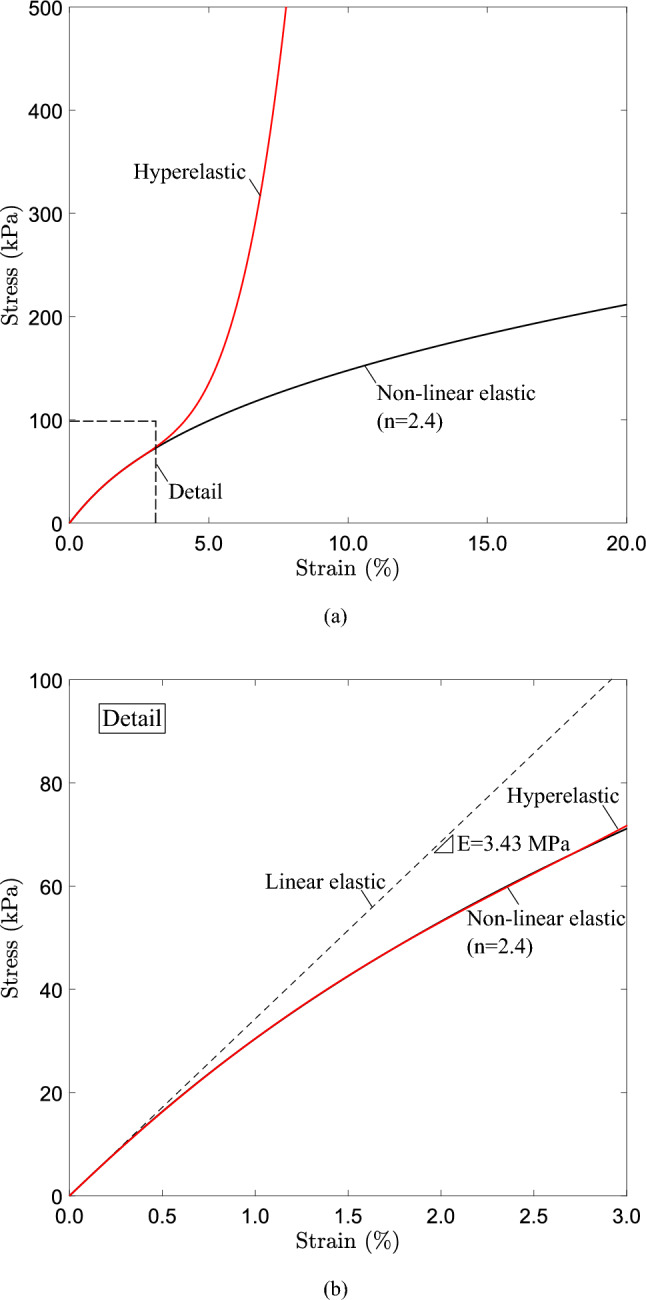
Stress–strain curves under uniaxial tension ($E=3.43$ MPa, $\nu =0.4$) **a** linear elastic, nonlinear elastic material ($n=2.4$) and Marlow hyperelastic material; **b** detail of **a** corresponding to strain level up to $3 \%$ where maximum pressure ($P_{max}$) occurs

## Description of finite element models

Finite element models are developed in Abaqus/Standard (Systèmes 2021) to simulate collapse and its propagation in long tubes subjected to uniform external pressure. Two types of models are considered. The first is a two-dimensional “ring” finite element model which represents a slice of an infinite-length tube under external pressure, free of boundary conditions, assuming that the tube is an assembly of consecutive rings which collapse in a sequential manner, as shown in Fig. 1b. Therefore, the propagation phenomenon can be described by the cross-sectional deformation of a single slice (ring) of the long tube, as shown in Fig. 1c. The second is a three-dimensional model, which simulates in a rigorous manner tube response in terms of collapse and propagation under uniform external pressure. Both numerical models account for nonlinear material properties, to be presented in Sect. 2.3. The analysis is static, aimed at tracing equilibrium diagrams of external pressure variation in terms of characteristic tube deformation measures.

**Fig. 4:**
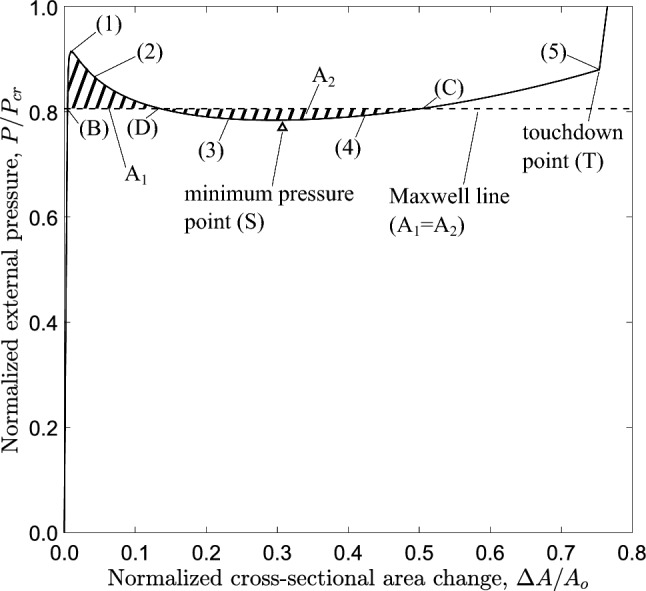
Diagram of external pressure versus area change from a collapsing ring model with $D/t=20$ and $n=2.4$ (symbol $\bigtriangleup$ indicates the point with minimum pressure value)

**Fig. 5:**
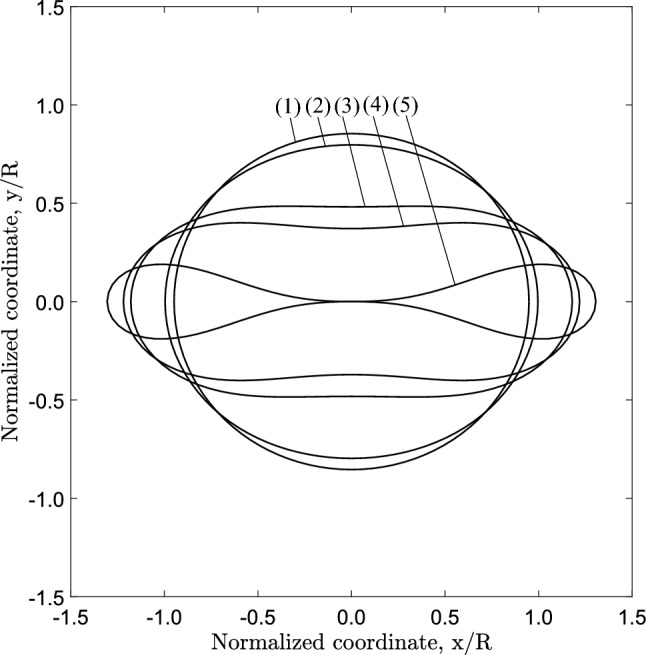
Deformed cross-sectional configurations at different stages of the post-buckling range of the collapsing ring obtained from the ring model ($D/t=20$ and $n=2.4$)

**Fig. 6:**
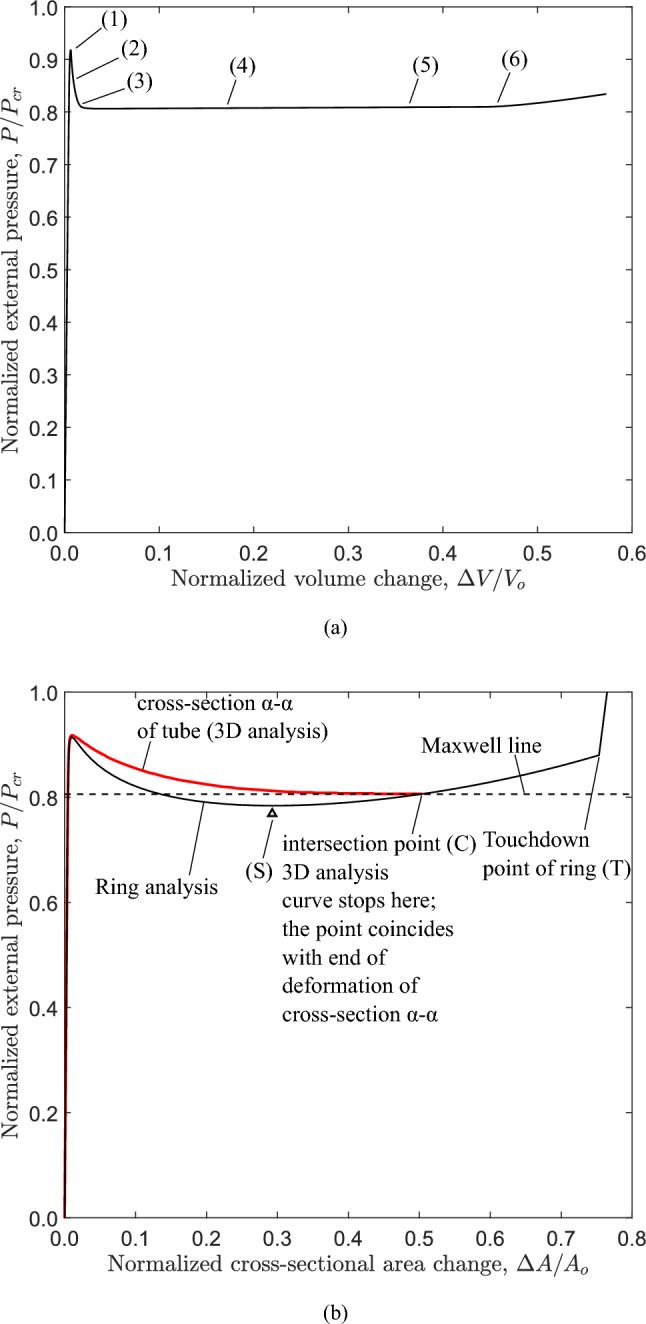
Diagram of external pressure versus: **a** volume change obtained from the 3*D* tube model and **b** area change from ring analysis and 3*D* analysis of cross section $\alpha$-$\alpha$ of the 3*D* tube model with the Maxwell line (see also Figure 7); $D/t=20$ and $n=2.4$

**Fig. 7:**
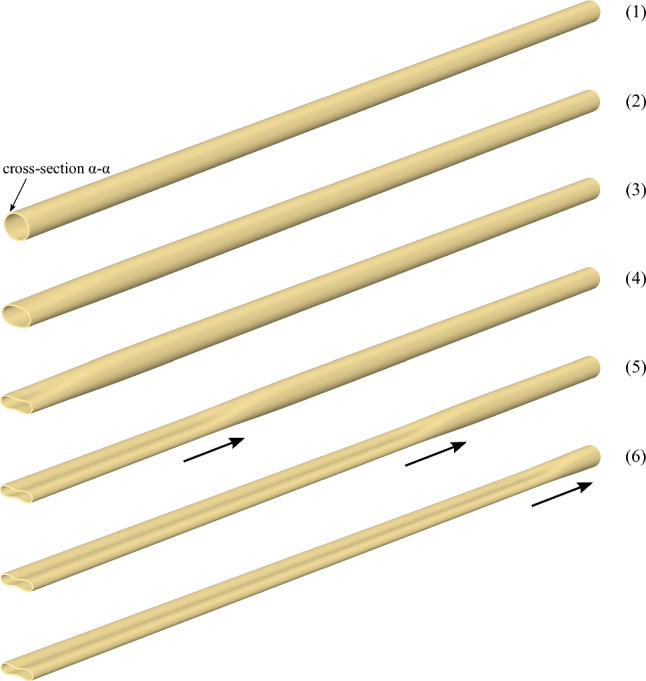
Deformation sequence of a long tube with $D/t=20$ and $n=2.4$ from buckle initiation at cross section ($\alpha$-$\alpha$) to steady-state buckle propagation

**Fig. 8:**
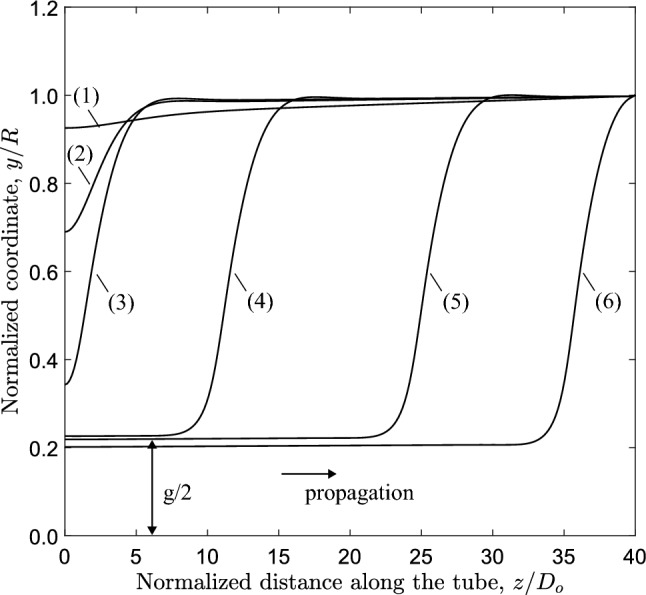
Configurations of the most deformed (top) generator during buckling propagation and residual gap; numbers (1–6) correspond to the deformed shapes of Fig. 7 ($D/t=20$, $n=2.4$)

### Ring model

The tube slice (ring) is shown in Fig. 2a and is loaded under uniform external pressure on its outer surface. The model geometry is defined by the outer diameter $D_{{{\text{o}}}}$, the wall thickness t, and a small length, equal to $10 \%$ of the outer diameter ($L=0.1D_{{{\text{o}}}}$). A doubly symmetric initial imperfection of the ring in the form of initial ovality is assumed on the $x-y$ plane (Fig. 2a) expressed as follows:

2 $$\begin{aligned} \frac{w_{o}\left( \theta \right) }{R}= \Delta _{o}\cos {(2\theta )} \end{aligned}$$

where $w_{o}$ is the radial deviation from the circular configuration, and $\Delta _{o}$ is the amplitude of initial imperfection, as shown schematically in Fig. 2b. It is equal to $0.2 \%$ and it is uniform from $z=0$ to $z=L$.

A quarter of the tube cross section is modeled because of double-symmetry. Symmetric boundary conditions with respect to *z* coordinate are applied on the edge nodes of $x-y$ plane at both ends, and on the bottom and the upper edge nodes of $y-z$ and $x-z$ planes. The tube slice is discretized with 20-node reduced-integration solid finite elements (denoted as C3D20R in Abaqus finite element library), with 25 elements in the circumferential direction, 3 elements in the radial (through-thickness) direction and 1 element in the longitudinal direction of the ring, as shown in Fig. 2a.

**Fig. 9:**
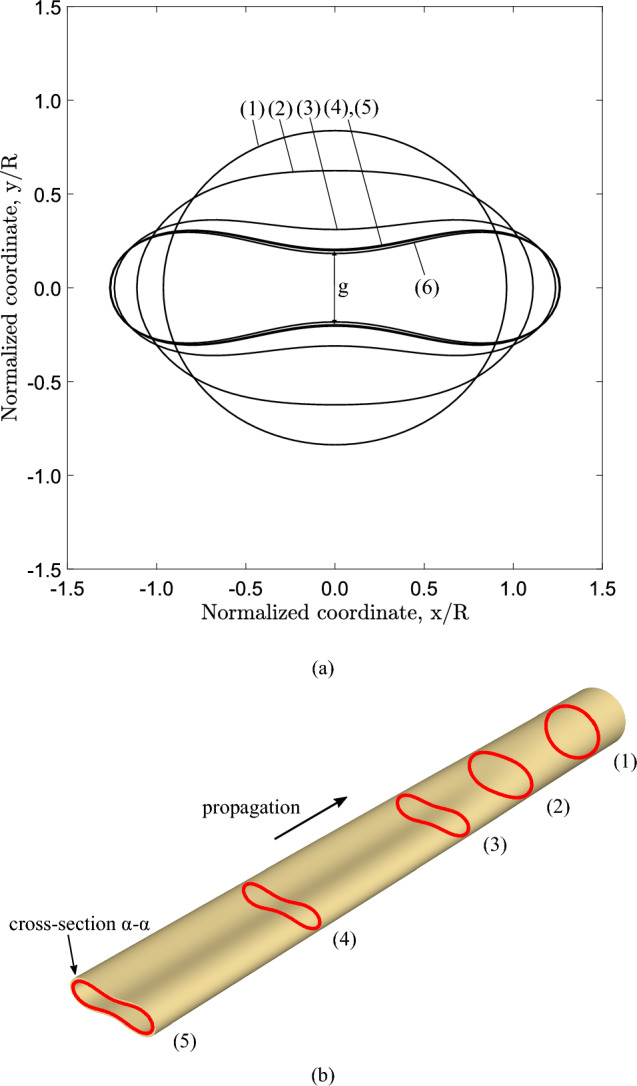
**a** Deformed configurations of cross section ($\alpha$-$\alpha$) at different stages in the post-buckling range for a tube with $D/t=20$ and $n=2.4$; **b** deformed configuration of the tube at quasi-static steady-state buckling propagation, represented by sequentially deformed rings, corresponding to the deformation stages, as shown in Fig. 9a

**Fig. 10:**
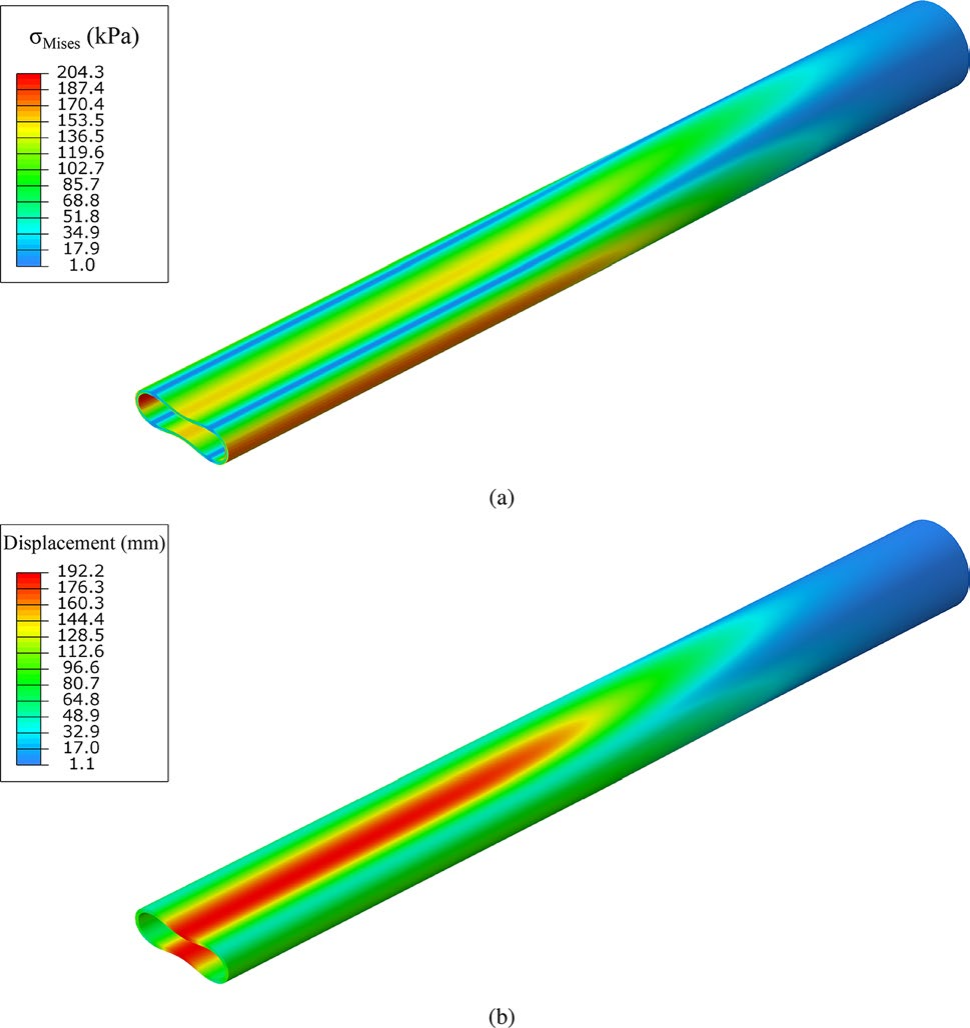
Deformed configuration of the tube with $D/t=20$ and $n=2.4$, showing **a** the von Mises stress distribution and **b** the field of total displacement

**Fig. 11:**
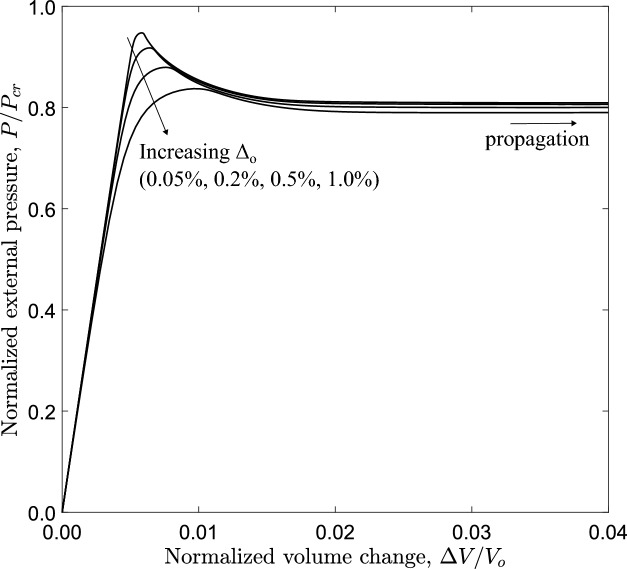
Diagram of external pressure versus volume change obtained from the 3*D* tube model, considering different initial ovalization ($\Delta _{o}$) values (0.05%, 0.2%, 0.5% and 1.0%); $D/t=20$ and $n=2.4$

**Fig. 12:**
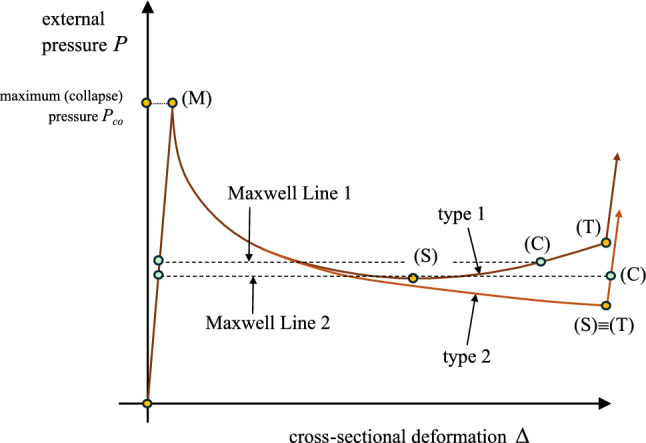
Schematic representation of ring response under external pressure and Maxwell line construction; point (C) is the intersection of Maxwell line with the ring collapse equilibrium diagram in type 1 response, point (C) is before touchdown point (T), whereas in type 2 response, point (C) occurs after touchdown point (T)

Uniform external pressure is applied through a nonlinear incremental static analysis that employs the modified Riks method, suitable for tracing unstable post-buckling equilibrium paths after a maximum load is reached. The post-buckling shape of the cross section is initially oval, followed by a “dog-bone” shape and continues to deform, until the upper and lower part of its inner surface of the tube establish contact. Contact is accounted for using an analytical rigid surface, at $x-z$ plane ($y=0$) as shown in Fig. 2a. A master–slave contact pair is created between the inner surface of the model (“slave”) and the analytical rigid surface (“master”) using frictionless surface-to-surface interaction and the “direct” method algorithm for “hard pressure-overclosure contact”.

### Three-dimensional (3*D*) tube model

To simulate in a rigorous manner tube collapse and its quasi-static propagation under external pressure, a three-dimensional finite element model is developed that replicates the response of a long tube. The finite element methodology developed by Gavriilidis et al. (2024) for offshore pipelines constitutes the basis for the present model. This methodology has been proven suitable for simulating the entire phenomenon and predicting satisfactorily the collapse pressure and the propagation pressure, compared with experimental tests. The finite element model is shown in Fig. 2c. Its length is equal to 40 outer diameters ($L=40D_{{{\text{o}}}}$) to allow for full development of the propagation phenomenon. An initial imperfection in the form of cross-sectional ovality is assumed, which varies along the *z* axial direction according to the following formula:

3 $$\begin{aligned} \frac{w_{o}\left( \theta ,z\right) }{R}= \left( 1-\frac{z}{L}\right) \Delta _{o}\cos {(2\theta )} \end{aligned}$$

where $\Delta _{o}$ is the amplitude of initial imperfection, taken equal to $0.2 \%$, which decays linearly from $z=0$ to $z=L$ so that at $z=L$ the cross section is a perfect cycle. Symmetric boundary conditions are imposed to the nodes at $z=0$ with respect to the *z* axis, while fixed boundary conditions are applied at $z=L$. Symmetric boundary conditions are applied on the edge nodes with respect to the $y-z$ and $x-z$ plane. The tube is modeled with 20-node reduced-integration solid finite elements (C3D20R in Abaqus). The mesh density in the circumferential and radial direction is the same with the one used for the ring model, whereas 250 finite elements are used in the longitudinal direction.

**Fig. 13:**
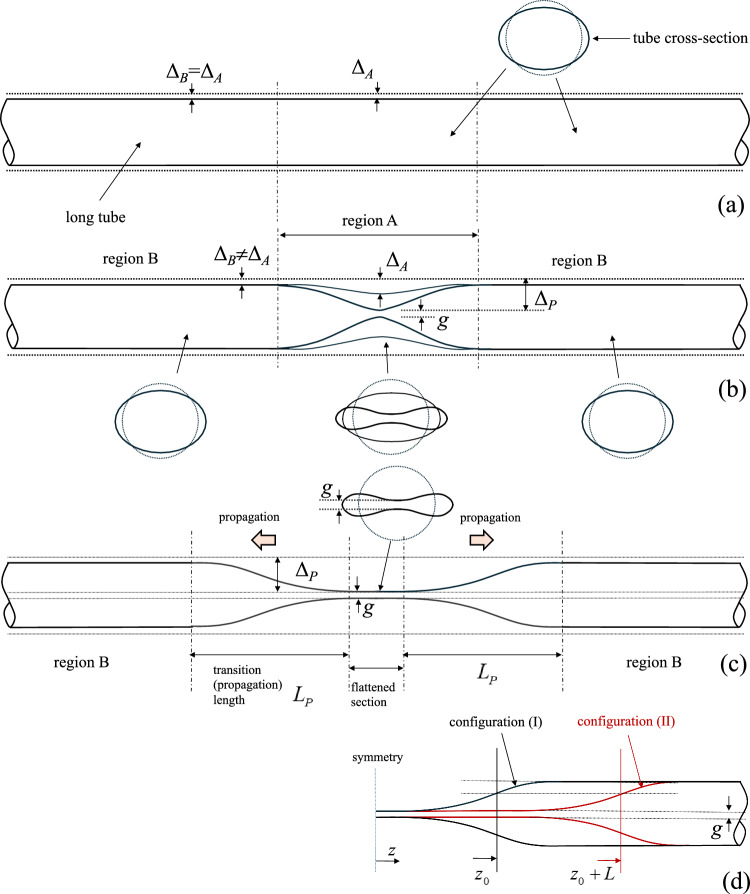
Schematic representation of deformation history in a long tube under external pressure; **a** pre-buckling stage with small uniform cross-sectional deformation, **b** localization of cross-sectional deformation in region *A*, **c** propagation stage and **d** buckle propagation by length L from stage (I) to stage (II)

**Fig. 14:**
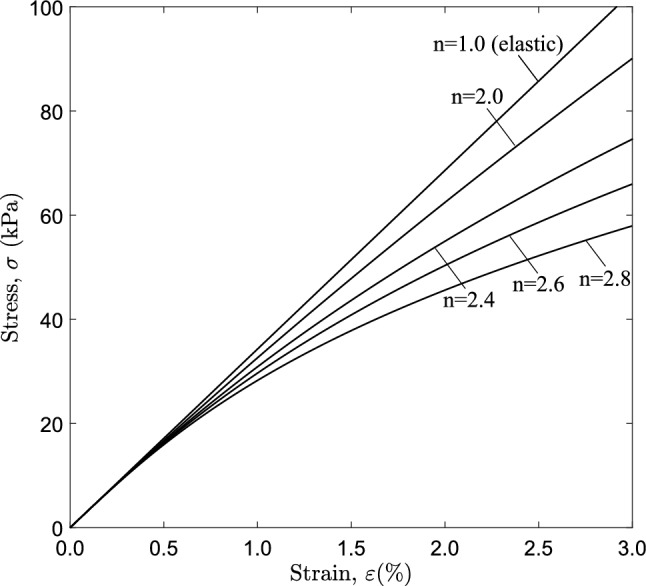
Stress–strain material curves for linear ($n=1.0$) and nonlinear elastic materials ($n>1$) for different values of exponent in eq. (4) and for strain up to $3 \%$

**Fig. 15:**
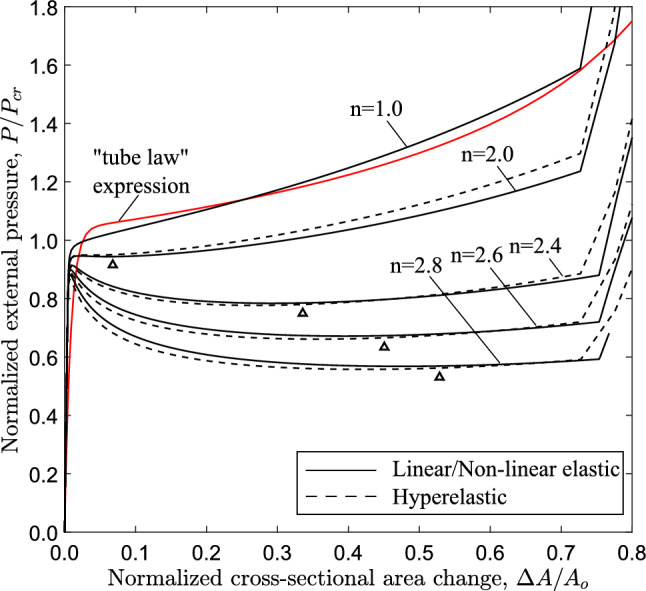
Diagram of external pressure versus area change (ring model) for a tube with $D/t=20$ and for $1\le n \le 2.8$; symbol $\bigtriangleup$ denotes the minimum pressure point on the diagram (“stiffening point”); comparison of the linear elastic case ($n=1.0$) with the analytical expression for the “tube law” proposed by Kozlovsky et al. (2014)

**Fig. 16:**
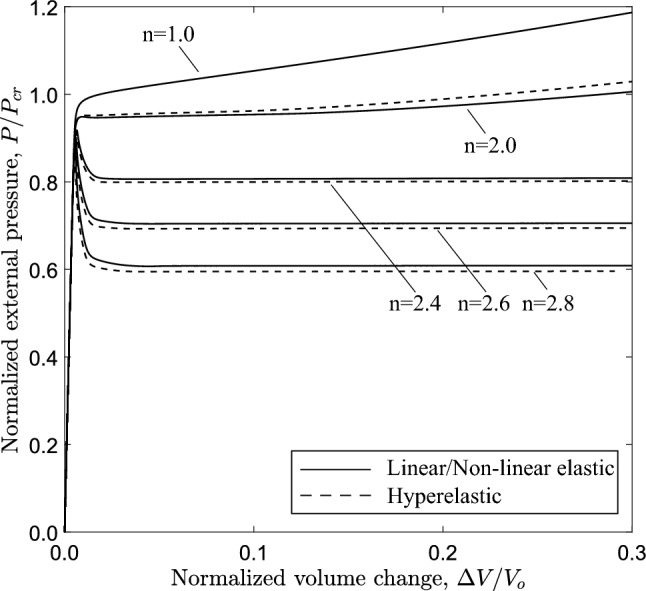
Diagram of external pressure versus volume change (3*D* model) for a tube with $D/t=20$, with different values of material exponent; the tube with $n=1.0$ collapses uniformly, without localization or propagation

The tube is surrounded by uniform external pressure, which is applied on its outer surface and raised incrementally using modified Riks method. Possible contact of the top and bottom part of the inner surface after complete collapse is modeled using an analytical rigid surface at $x-z$ plane, extended along the entire length of the model as shown in Fig. 2c. The contact algorithm is the same with the one considered in the ring model.

### Material modeling

Biological conduits are highly flexible and deformable and their material can be considered as nonlinear elastic or “rubber-like” and its mechanical behavior has been described through phenomenological models either hyperelastic (Capurro and Barberis 2014; Zhu et al. 2008; Kozlovsky et al. 2014) or nonlinear elastic (Heil and Pedley 1996; Heil 1997; Marzo et al. 2005). Therefore, the effect of material softening on the structural behavior of tubes under external pressure is examined considering nonlinear elastic material properties which deviate from linear elastic behavior. Two different constitutive models are employed; the $J_{2}$ deformation theory model, and Marlow hyperelastic model, which are briefly described as follows.

The $J_{2}$ deformation theory model is included in Abaqus/Standard (Systèmes 2021) and is essentially a path-independent nonlinear elastic model. Under uniaxial stress conditions, the strain $\left( \varepsilon \right)$ and the stress $\left( \sigma \right)$ are related through the following relation:

4 $$\begin{aligned} \varepsilon = \frac{\sigma }{E} \left[ 1 + \alpha _{o} \left( \frac{\sigma }{\sigma _{o}}\right) ^{n-1}\right] \end{aligned}$$

where *E* is Young’s modulus, $\sigma _{o}$ is a stress parameter, $\alpha _{o}$ is the limit offset coefficient and *n* is the exponent that characterizes nonlinearity. In the following, this model will be referred to as “nonlinear elastic model”. One may note that for $n=1.0$ the nonlinear elastic model of eq. (4) turns into a linear elastic model.

**Fig. 17:**
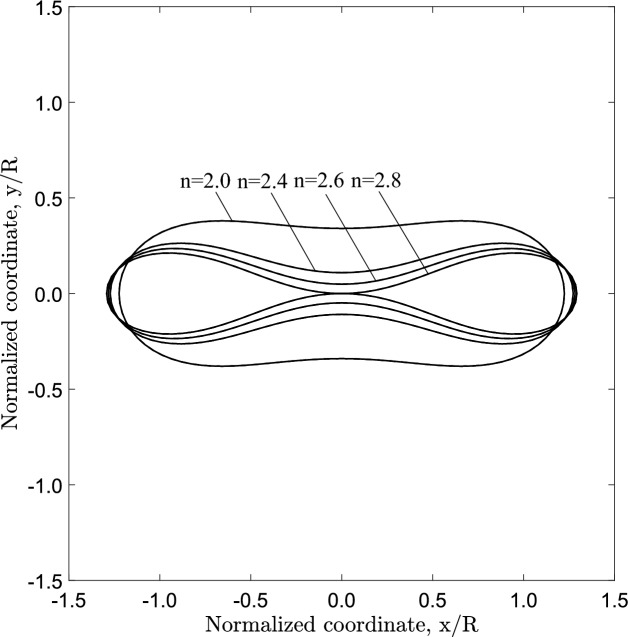
Configuration of the propagation profile of a cross section under steady-state conditions for a tube with $D/t=20$ and different levels of material nonlinearity; contact is established only for $n=2.8$

**Fig. 18:**
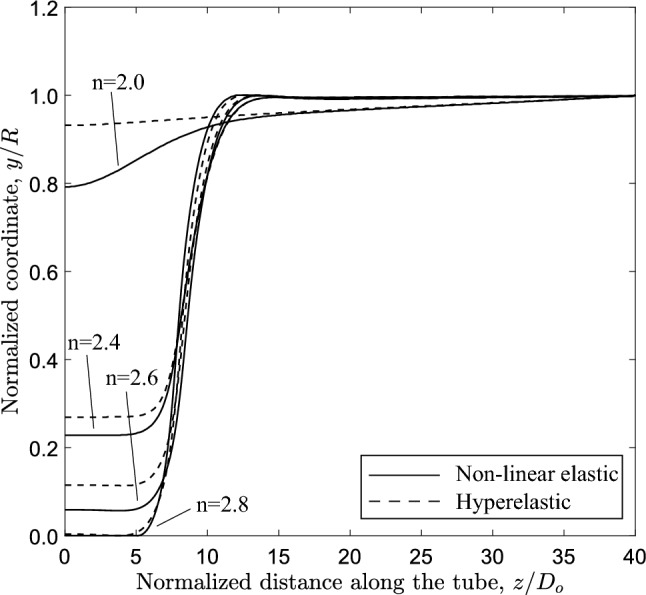
Configuration of the most deformed tube generator (top generator) during propagation for different levels of material nonlinearity ($n=2.0, 2.4, 2.6, 2.8$); contact is established only for $n=2.8$

**Fig. 19:**
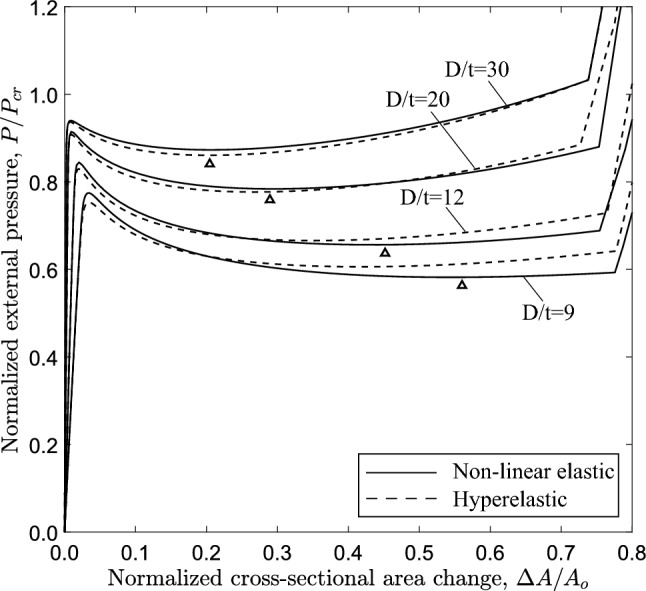
External pressure versus area change (ring model) for elastic tubes of different *D*/*t* ($n=2.4$)

**Fig. 20:**
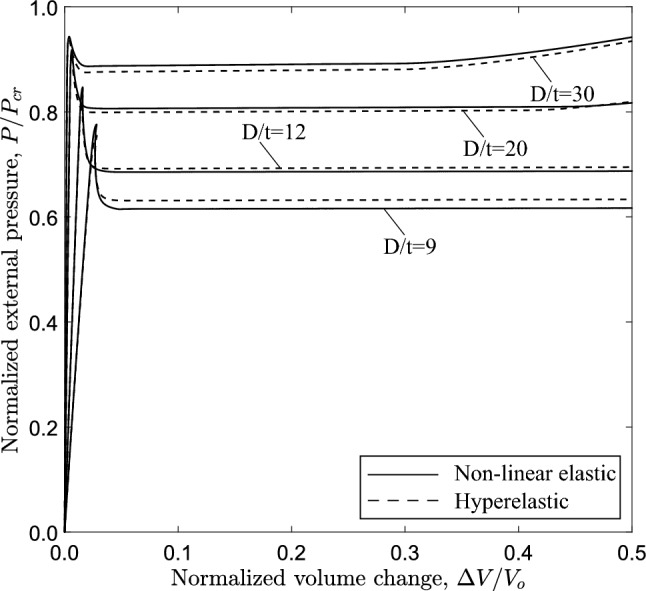
External pressure versus volume change diagram (3*D* model) for elastic tubes of different *D*/*t* ($n=2.4$)

**Fig. 21:**
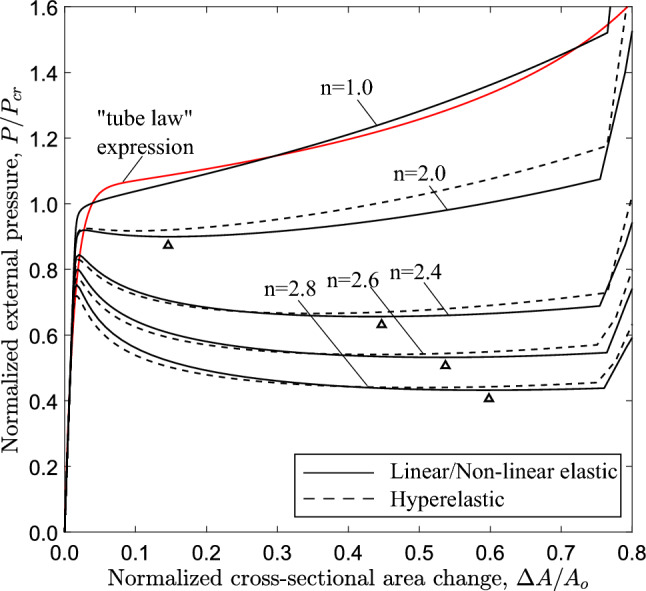
External pressure versus area change diagram (ring model) for a tube with $D/t=12$; comparison of the linear elastic case ($n=1.0$) with the “tube law” analytical expression proposed by Kozlovsky et al. (2014)

**Fig. 22:**
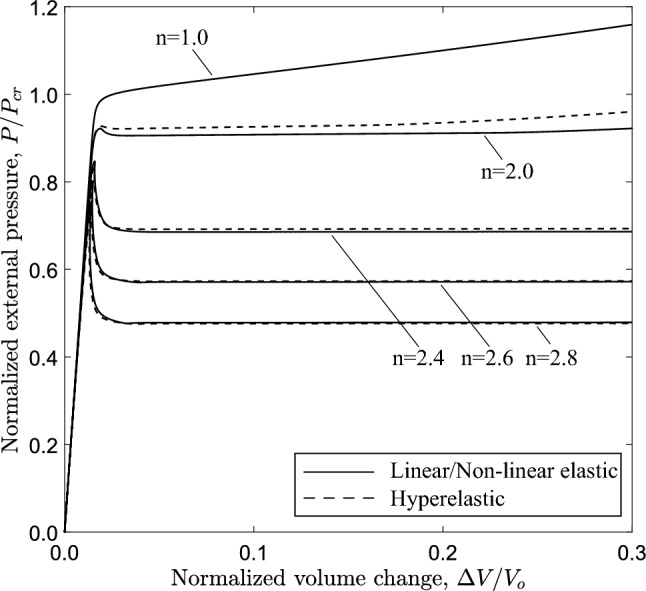
Diagram of external pressure versus volume change (3*D* model) for a tube with $D/t=12$

**Fig. 23:**
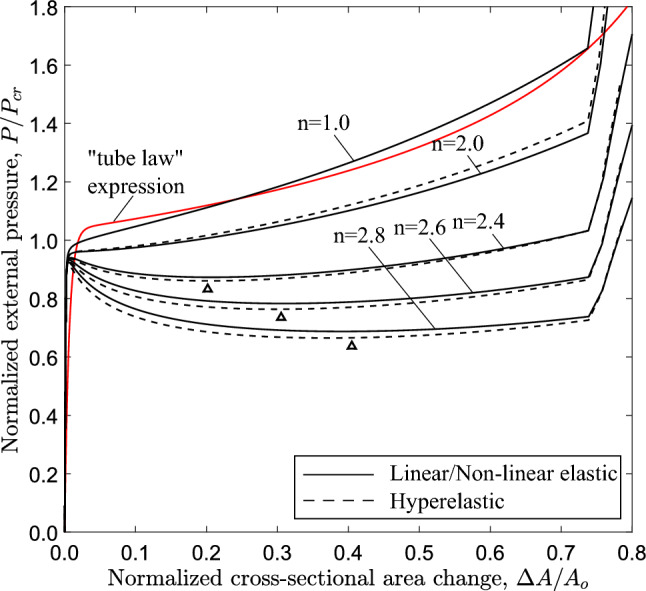
External pressure versus area change diagram (ring model) for a tube with $D/t=30$; comparison of the linear elastic case ($n=1.0$) with the “tube law” analytical expression proposed by Kozlovsky et al. (2014)

**Fig. 24:**
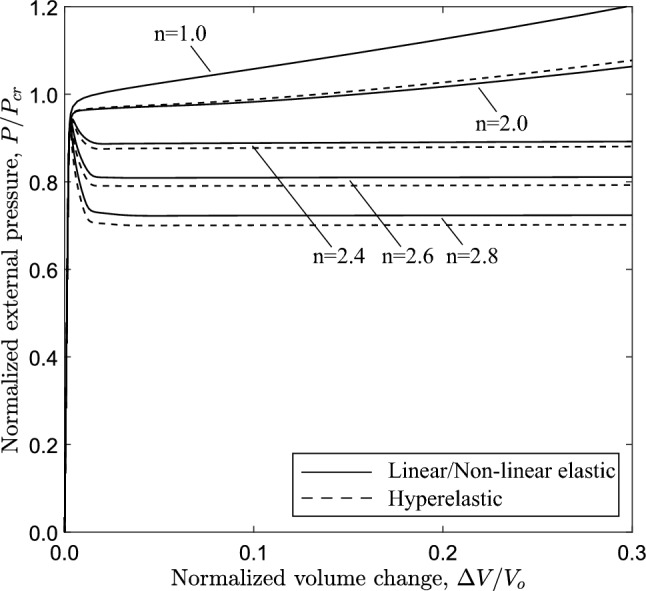
Diagram of external pressure versus volume change (3*D* model) for a tube with $D/t=30$

**Fig. 25:**
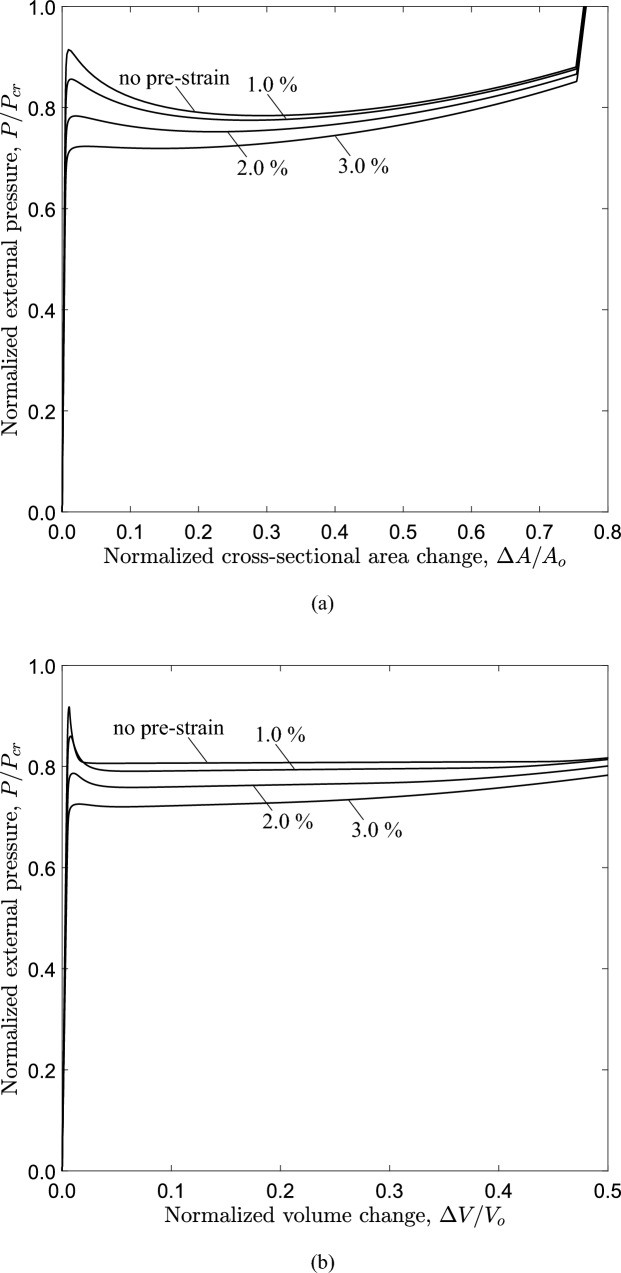
Equilibrium diagrams of **a** external pressure versus area change obtained from the ring model and **b** external pressure versus volume change obtained from the 3*D* model for three levels of axial pre-strain; $1.0\%$, $2.0 \%$ and $3.0\%$ ($D/t=20$ and $n=2.4$)

**Fig. 26:**
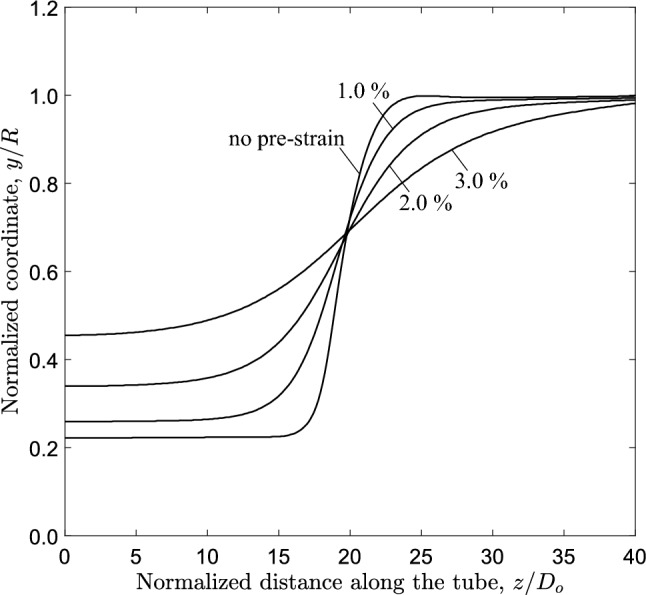
Configuration of the most deformed generator of 3*D* tube model with *D*/*t* ratio equal to 20 and *n* equal to 2.4 for three levels of axial pre-strain; $1.0\%$, $2.0 \%$ and $3.0\%$

**Fig. 27:**
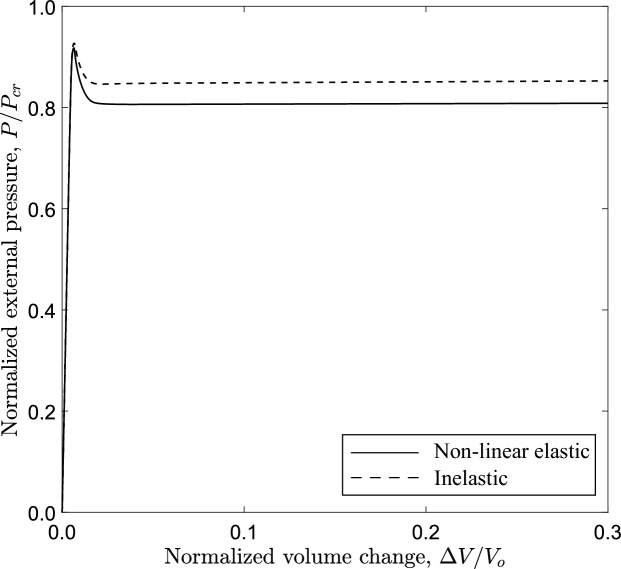
Diagram of external pressure versus volume change for a tube with $D/t=20$, $n=2.4$; 3*D* model analysis

The hyperelastic stress–strain material curve, as shown in Fig. 3 for uniaxial tension conditions, follows Marlow material model (Marlow 2003), which is included in Abaqus. The main feature of the Marlow model is its ability to build the strain energy potential (*U*) by integrating the stress–strain data that define the deviatoric and the volumetric material behavior (Marlow 2003). The model belongs to the family of first-invariant hyperelastic constitutive models, and is expressed in terms of the modified first invariant $\bar{I}_1$ of the right Cauchy-Green strain tensor and the Jacobian determinant *J* of the deformation gradient (Marlow 2003; Systèmes 2021; Hüter and Rieg 2021):

5 $$\begin{aligned} U = U_{d}(\bar{I}_1)+ U_{v}(J) \end{aligned}$$

where $U_{d}(\bar{I}_1)$ and $U_{v}(J)$ are the deviatoric part and the volumetric part of the strain energy potential, respectively. Function $U_{d}(\bar{I}_1)$ is determined by integrating the stress data $\sigma (\varepsilon )$:

6 $$\begin{aligned} U_d(\bar{I}_1) = \int _0^\varepsilon \sigma (\varepsilon ) \, {\text{d}}\varepsilon \end{aligned}$$

Function $U_{v}(J)$ of eq. (5) refers to the volumetric part of material response and is defined, taking into account the compressibility of the material (Marlow 2003; Systèmes 2021; Hüter and Rieg 2021). The advantages of Marlow model over other available first-invariant constitutive models stem from eq. (6), where the stress–strain response under a given deformation mode (e.g., uniaxial tension) is exactly reproduced in a very straightforward manner (Hüter and Rieg 2021). Additionally, the model is very efficient when limited material data are available; for example, when stress–strain data from uniaxial tension are only available.

Figure 3a shows the comparison of uniaxial behavior between these material models for $n=2.4$. The initial part of the stress–strain diagram up to $3 \%$ strain is associated with the maximum pressure and initial post-buckling response of the elastic tubes examined herein. Specifically, the material softening represented by the deviation of the diagram from the linear elastic curve (also shown in Fig. 3b) is of particular interest in the present analysis. The stress–strain curves shown in Fig. 3 refer to nominal stress and engineering strain; they may be expressed in terms of true stress $\sigma _{t}$ and logarithmic strain $\varepsilon _{t}$, using the well-known analytical expressions; $\sigma _{t}=\sigma (1+\varepsilon )$, $\varepsilon _{t}=ln(1+\varepsilon )$. Abaqus considers true stress–strain data and thus large deformation is taken into account. In all cases, Young’s modulus *E* and Poisson’s ratio $\nu$ of the material are $E=3.43$ MPa and $\nu =0.4$, which are typical values for biological tubes (Kozlovsky et al. 2014; Silver et al. 2021). The other parameters of the nonlinear elastic model used in the present study are $\sigma _{o}=4$ kPa, $\alpha _{o}=6.31$ x $10^{-3}$ and exponent *n* varies between 1 and 2.8. The stress–strain data obtained by eq. (4) up to strain $3 \%$ are used to calibrate the Marlow model, and the corresponding stress–strain curve is shown in Fig. 3b, exhibiting a first softening branch and a subsequent hardening part, typical of a hyperelastic material.

## Numerical results for tubes with $D/t=20$ and $n=2.4$

The stability of externally pressurized tubes with $D/t=20$ and nonlinear elastic material behavior corresponding to $n=2.4$ (shown in Fig. 3) is analyzed with the finite element methodology presented in the previous section. The numerical results offer an initial ground for discussion of several key features of collapsible tube behavior and are presented in terms of pressure-deformation diagrams and deformed tube configurations.

### Ring model analysis

Performing a nonlinear static analysis with incremental application of pressure on the outer surface of the tube slice (Fig. 2a), the equilibrium diagram of Fig. 4 is obtained. The change of area enclosed by the tube cross section, $\Delta A$, is normalized by the initial area enclosed by the tube cross section ($A_{o}$), and the applied external pressure (*P*) is normalized by the elastic critical buckling pressure $P_{{{\text{cr}}}}$ calculated by eq. (1). The response indicates a quasi-linear path until the external pressure reaches a peak value [stage “(1)”], which is the “collapse pressure”, $P_{{{\text{co}}}}$. After maximum pressure is reached, the structural stiffness becomes negative and the pressure is substantially reduced as indicated by the descending post-buckling curve of the response. Figure 5 shows the deformed configurations corresponding to stages $(1)-(5)$ in Fig. 4. Because of the oval shape in the post-buckling regime excessive deformation occurs at four equally spaced circumferential locations where high bending strains are developed, and due to material softening the local bending rigidity of the ring at those locations is reduced. The unstable post-buckling branch reaches a minimum pressure of the “stiffening” point (S), indicated by the $\bigtriangleup$ symbol, and a stable response with increasing pressure follows beyond that point. Eventually, the cross section obtains a “dog-bone” shape (stage “(5)” in Fig. 5), and contact is established at point (T) between the upper and lower side of the inner surface of the ring, resulting in a sharp increase of pressure.

The intermittent line shown in Fig. 4 that makes areas $A_{1}$ and $A_{2}$ equal, follows the Maxwell line construction method, described in the work of Chater and Hutchinson (1984) and constitutes an estimate of the propagation pressure. This method is based on energy balance, and specifically on the argument that the difference in internal energy between two stable branches, where the pressure increases, being separated by an unstable branch, where the pressure decreases can be used to calculate the propagation pressure, $P_{{{\text{p}}}}$, under the assumption of quasi-static propagation (Kyriakides and Lee 2020; Karamanos 2022). Considering path-independent material behavior, the internal energy $\hat{U}_{r}$ dissipated by the collapsing ring during propagation should be equal to the external work $\hat{W}_{\text{ext}}$ required to jump from point “(B)” on the pre-buckling stable branch to point “(C)” on the second stable branch. For the collapsing ring, the internal energy dissipated between points “(B)” and “(C)”, as shown in Fig. 4, is expressed by the following equation:

7 $$\begin{aligned} \hat{U}_{r}=\int _{\Delta A_{\text{B}}}^{\Delta A_{C}} P~d\Delta A \end{aligned}$$

The work of external pressure $\hat{W}_{\text{ext}}$ between the two corresponding locations is expressed by the following equation:

8 $$\begin{aligned} \hat{W}_{\text{ext}}= P_{{{\text{p}}}} (\Delta A_{C}-\Delta A_{\text{B}}) \end{aligned}$$

Assuming path-independent material behavior, which is always the case in elastic materials, balance of energy requires that $\hat{U}_{r}=\hat{W}_{\text{ext}}$. Equating the right hand sides of eq.(7) and eq.(8), the propagation pressure is calculated as follows:

9 $$\begin{aligned} P_{{{\text{p}}}} =\frac{1}{\Delta A_{C}-\Delta A_{\text{B}}}\int _{\Delta A_{\text{B}}}^{\Delta A_{C}} P~d\Delta A \end{aligned}$$

The propagation pressure value in eq. (9) is graphically represented by the Maxwell line construction, which corresponds to the horizontal line that makes areas $A_{1}$ and $A_{2}$ equal, as depicted in Fig. 4. This method results to a normalized value for the propagation pressure ($P_{{{\text{p}}}} / P_{{{\text{cr}}}}$) equal to 0.809, or equivalently $P_{{{\text{p}}}} =0.823$ kPa. It is interesting to note that, for the tube under consideration, the stable stage “(C)” corresponds to a ring configuration, before the top and bottom part of the inner surface of the ring wall establish contact, as opposed to metal rings (Kyriakides and Lee 2020; Karamanos 2022).

### Three-dimensional (3*D*) tube model analysis

Figure 6a presents the equilibrium diagram of pressure *P* in terms of the volume change $\Delta V$ enclosed by the tube under consideration ($D/t=20$ and $n=2.4$). Again, the value of external pressure (*P*) is normalized by the elastic critical buckling pressure ($P_{{{\text{cr}}}}$) of eq. (1), and the value of $\Delta V$ is normalized by the initial volume enclosed by the tube ($V_{o}$). In each increment during the analysis, the value of $\Delta V$ is calculated by an external post-processing tool, developed in-house, which employs the node coordinates of the deformed inner surface of the tube.

The numbers in brackets on the pressure-volume diagram of Fig. 6a correspond to the deformed configurations of the long tube shown in Fig. 7. The pressure-volume diagram shows that the tube is initially stiff and the pressure increases rapidly to a pressure maximum of 0.937 kPa [stage “(1)”, $P/ P_{{{\text{cr}}}} =0.921$)]. This corresponds to the onset of local collapse of the tube at $z=0$ (cross section $\alpha$-$\alpha$). Upon reaching this maximum value, the tube becomes highly unstable and the post-buckling deformation is characterized by rapid drop of pressure and excessive ovalization of the cross section at $z=0$ [stage “(2)”]. Eventually, a minimum value of pressure is reached at stage “(3)”, after which the collapsed configuration at cross section $\alpha$-$\alpha$, often referred to as “buckle”, starts propagating along the tube under constant external pressure 0.824 kPa ($P/ P_{{{\text{cr}}}} =0.811$), represented by stages “(4)” and “(5)” in Fig. 6a. The corresponding deformed configurations of the tube during buckle propagation are shown in Fig. 7. It is important to notice that the buckle propagates without contact of the upper and lower side of the inner surface of the tube, forming a gap $g=0.11R_{o}$ (or $g=0.22D_{{{\text{o}}}}$), and this is shown very clearly in Fig. 7 at stages “(4)” and “(5)”. During propagation, the pressure remains constant, equal to $P_{{{\text{p}}}}$, and begins to rise only when the buckle approaches the fixed boundary condition of the tube at $z=L$ [stage “(6)”]. Furthermore, the value of $P_{{{\text{p}}}}$ is lower than the value of the collapse pressure $P_{{{\text{co}}}}$.

The thick red line in Fig. 6b shows the $P-\Delta A$ diagram for the cross section of the long tube at $z=0$ (section $\alpha$-$\alpha$) obtained from the 3*D* analysis. This diagram stops at $\Delta A=0.51$ which corresponds to the cross-sectional configuration during steady-state propagation, and compares well with the *P*-$\Delta A$ diagram of Fig. 4, from ring analysis, also plotted in Fig. 6b for convenience. The small difference in the initial post-buckling response of the two diagrams is attributed to longitudinal stretching of the tube generators. Nevertheless, the overall comparison of the two curves is very good. It is very interesting to notice that the 3*D* analysis curve stops exactly at the point where the Maxwell line intersects the *P*-$\Delta A$ curve of the ring analysis, an issue to be discussed in Sect. 4 of this paper.

The noncontact configuration of the tube during propagation is also shown in Fig. 8 that depicts the shape of the most deformed generator of the tube at $y-z$ plane, for the deformed configurations presented in Fig. 7. Localization of collapse starts at stage (1) just after the maximum pressure, and subsequently cross-sectional deformation at $z=0$ increases [stages (2) and (3)] forming the “buckle”. The subsequent profiles at stages (2), (3), (4) and (5) show the steady-state propagation, while the final size of the gap (*g*) between the two surfaces is $22\%$ of the tube outer diameter. The deformed cross-sectional configurations corresponding to stages $(1)-(6)$ at $z=0$ are shown in Fig. 9a, and the configuration of the collapsing tube interpreted as a sequence of progressively deformed rings is shown in Fig. 9b. Comparison of the final shape of the tube cross section (Stage 5) in Fig. 9a with the corresponding shape in Fig. 1c shows that the shape of the elastic–plastic tube is sharper at locations $\theta =0$, $\pi /2$, $\pi$, $3 \pi /2$ because of the presence of inelastic deformation at those locations. The collapse pressure values from the three-dimensional model (0.937 kPa) and the ring model (0.934 kPa) are in a good agreement. Additionally, the propagation pressure values obtained from the 3*D* model and the ring model are 0.824 kPa and 0.823 kPa, respectively, sharing good agreement as well. Figure 10a presents the von Mises stress distribution at the propagation profile, while Fig. 10b depicts the corresponding total displacements. The results show that the maximum von Mises stress, approximately 205 kPa, is developed at the four equally spaced locations around the circumference with maximum bending.

The effect of imperfection amplitude $\Delta _{o}$ on the collapse and buckling propagation is also examined for the tube under consideration ($D/t=20$ and $n=2.4$). Figure 11 shows the normalized equilibrium diagram of pressure in terms of the volume change for $\Delta _{o}$ equal to $0.05\%$, $0.2\%$, $0.5\%$ and $1.0\%$. The results show that increasing the initial imperfection from $0.05\%$ to $1.0\%$ the collapse pressure is reduced by $12.0\%$, while the influence of the $\Delta _{o}$ value on propagation pressure is negligible.

## Localization and propagation mechanism

To enlighten the mechanism of “propagating buckles”, a simple and efficient analytical model is employed. In its early form, the model was introduced by Tvergaard and Needleman (1980), for explaining the development of localized buckling patterns in structural systems and the onset of material instability (e.g., necking) under severe tensile loading. The model is considered herein in an enhanced form, suitable for the case of buckling propagation in collapsible tubes.

The tube is considered as an assembly of deformable rings (Fig. 1b). Each ring is assumed to have the structural response shown in Fig. 12, in terms of the equilibrium diagram of external pressure versus ring deformation. In mathematical terms, one may write the following incremental equilibrium equation for the deforming ring:

10 $$\begin{aligned} \dot{P}=K\dot{\Delta } \end{aligned}$$

where *P* is the externally applied pressure, $\Delta$ is a measure of deformation, usually its cross-sectional ovalization or the change of area enclosed by the ring, $\dot{()}$ denotes differentiation with respect to a time-like parameter and *K* is the instantaneous structural stiffness of the ring,

11 $$K = \frac{{{\text{d}}P}}{{{\text{d}}\Delta }}$$

At early stages of pressure loading, the $P-\Delta$ response of the ring is quasi-linear, *K* is positive and cross-sectional deformation $\Delta$ is small and uniform along the tube (Fig. 13a). Subsequently, the possibility of bifurcation from this uniform deformation state ($P,\Delta$) to a localized pattern is sought, where the bifurcation mode consists of a localized region of the tube denoted as “region A” (Fig. 13b), which exhibits incremental deformation $\dot{\Delta }_{\text{A}}$ different from the one $\dot{\Delta }_{\text{B}}$ at the bulk of the tube denoted as “region B”.

Considering uniform external pressure around the entire tube, one may write:

12 $$P_{{\text{A}}} = P_{{\text{B}}} = P$$

where subscript A or B denotes region A or region B, respectively. Therefore,

13 $$\begin{aligned} \dot{P}= & K\dot{\Delta }_{\text{A}} \end{aligned}$$

14 $$\begin{aligned} \dot{P}= & K\dot{\Delta }_{\text{B}} \end{aligned}$$

Combining equations (13) and (14), one readily obtains:

15 $$\begin{aligned} K(\dot{\Delta }_{\text{A}}-\dot{\Delta }_{\text{B}})=0 \end{aligned}$$

Localization of deformation in region A requires that $\dot{\Delta }_{\text{A}}\ne \dot{\Delta }_{\text{B}}$, and therefore, one obtains from eq. (11):

16 $$K = \frac{{{\text{d}}P}}{{{\text{d}}\Delta }} = 0$$

In other words, localization of deformation along the tube is possible only when a maximum pressure value is reached on the $P-\Delta$ diagram, denoted as stage “(M)” in the schematic diagram of Fig. 12. This is verified by the numerical results in Figs. 6, 7, 8 and 9: the maximum pressure is followed by localization of deformation in region *A* shown schematically in Fig. 13b, the system becomes structurally unstable and a significant drop of pressure occurs.

The unstable post-buckling condition of the ring continues until resistance of the collapsing ring to further ovalization starts to develop again. This is referred to as “stiffening”, it starts at point “(S)” (shown in Fig. 12) and it is due to either local stretching of the most ovalized section (e.g., in the case examined in Sect. 3) before contact occurs at (*T*), or contact of two opposite sides of the inner surface due to complete ring collapse. In a three-dimensional tube, soon after reaching such a “stiffening” state in the most deformed cross section of region *A*, instead of further cross-sectional deformation and pressure increase, the long tube finds it easier to deform the adjacent cross section, shown schematically in Fig. 13c, until the adjacent cross section reaches a similar “stiffening” state. Repeatedly, a “steady-state” is reached, where each cross section enforces its adjacent one to deform, resulting in progressive collapse of the tube and in propagation of the phenomenon. In such a case, the length of region A increases and the collapsed pattern propagates, flattening the entire tube. It is of particular interest to notice in Fig. 6b that the deformation of a cross section in a 3*D* propagation analysis stops exactly at the point where the Maxwell line intersects the ring analysis curve (i.e., at $\Delta A=0.51$), and this demonstrates the strong correlation between the ring analysis and the buckle propagation phenomenon.

The steady-state propagation of the buckle by a length *L* is shown in the schematic representation of Fig. 13d. One may readily observe that translation of the buckle by a distance *L*, means that all cross sections within a length equal to *L* deform from circular to final configuration. Therefore, the strain energy required for translating the buckle profile by *L* (denoted as $E_{r}$), can be expressed as the product of the strain energy, $\hat{U}_{r}$, required for the collapse of a ring, i.e., a slice of the tube with unit length, multiplied by the amount of translation *L*. In mathematical terms:

17 $$\begin{aligned} E_{r}=\hat{U}_{r}L \end{aligned}$$

and $\hat{U}_{r}$ is calculated by eq. (7).

## Parametric study

The influence of material nonlinearity, *D*/*t* ratio and pre-strain level on the collapse pressure, propagation pressure and post-buckling behavior of elastic tubes are examined. Furthermore, the response of elastic tubes is compared with the response observed in tubes made of materials that exhibit inelastic (irreversible) deformation.

### Effect of material nonlinearity on collapse and propagation

The tubes analyzed in this section of the study have $D/t=20$ and initial imperfection amplitude $\Delta _{o}=0.2 \%$. Different values of exponent (*n*) are considered for the nonlinear elastic material, ranging from 1.0 (linear elastic) to 2.8, and the corresponding uniaxial stress–strain curves are presented in Fig. 14. The Marlow hyperelastic material model is also used, calibrated with data from the curves of Fig. 14 up to strain level of $3 \%$ (Fig. 3).

Figure 15 shows the variation of external pressure, with respect to the cross-sectional area change of the ring $\Delta A$, obtained with a ring analysis. For all cases, except for the linear elastic case ($n=1.0$), the response exhibits a pressure maximum, and beyond this maximum the pressure drops rapidly with a negative slope branch. The post-buckling response of the linearly elastic ring follows a stable loading path with increasing pressure. The tube law expression reported by Kozlovsky et al. (2014) is also included in Fig. 15, which is written below using the notation of the present paper:

18 $$\begin{aligned} \frac{P}{ P_{{{\text{cr}}}} }=C_{p}\left[ \left( 1-\frac{\Delta A}{A_{o}}\right) ^{m_{1}} -\left( 1-\frac{\Delta A}{A_{o}}\right) ^{m_{2}}\right] \end{aligned}$$

where the parameters $C_{p}$, $m_{1}$ and $m_{2}$ are given below:

19 $$\begin{aligned} \begin{aligned} C_{p}=0.129\left[ \left( \frac{D_{{{\text{o}}}} }{t}-1\right) ln\left( \frac{D_{{{\text{o}}}} }{D_{{{\text{o}}}} -2t}\right) \right] ^{3} \\ m_{1}=\frac{60}{\left( \frac{2t}{D_{{{\text{o}}}} -2t}\right) ^{0.5}} - 65 \\ m_{2}=0.7\left( \frac{2t}{D_{{{\text{o}}}} -2t}\right) - 0.4 \end{aligned} \end{aligned}$$

The tube law expression of eq. (18) compares very well with the linear elastic case ($n=1.0$). Increasing the nonlinearity of the elastic material (increasing the value of *n*), the maximum pressure reduces, and its normalized value is equal to 0.950, 0.918, 0.902 and 0.885 for exponent values 2.0, 2.4, 2.6 and 2.8, respectively, while the unstable part becomes more pronounced. It should be highlighted that in case of $n=2$, the maximum pressure is hardly detected in the graph, and this is the minimum value of exponent *n* resulting in unstable post-buckling response. For all cases except the linear elastic, a minimum pressure is reached in the unstable branch (“stiffening” point), while beyond that point the pressure increases. All curves exhibit a sharp increase of pressure once the top and the bottom of the tube wall establish contact. Figure 15 also depicts the corresponding equilibrium paths obtained from the ring model, with Marlow hyperelastic model, which are very close to those obtained with the nonlinear elastic material model.

The effect of material nonlinearity is also examined considering the three-dimensional tube model. Figure 16 presents the external pressure with respect to the volume change enclosed by the tube. For nonlinear elastic material with exponent $n=2$, 2.4, 2.6 and 2.8, a maximum pressure is reached, followed by unstable post-buckling response. Increasing the nonlinearity of the material (i.e., the nonlinearity exponent *n*), the maximum pressure is reduced and the unstable post-buckling behavior becomes more pronounced. Clearly, the case of $n=2.0$ constitutes the borderline between stable and unstable buckling response.

The configuration of the tube cross section under steady-state buckling propagation conditions is presented in Fig. 17 for different values of the exponent. The results show that contact of the upper and lower part of the tube wall is established only when $n=2.8$, whereas for *n* values up to 2.6, there is a gap between the inner surfaces of the collapsed configuration during buckle propagation, which decreases with increasing values of *n*. On the other hand, elastic tubes with linear or quasi-linear elastic material ($n<2$) do not develop propagating buckles, but collapse uniformly, a result consistent with observations in previous works on linear elastic tubes (Dyau and Kyriakides 1993). One may also note that buckle propagation with gap occurs when the Maxwell line in ring model analysis intersects the equilibrium path before the touchdown point, based on the results shown in Fig. 15. On the other hand, contact is established during propagation when the Maxwell line intersects the equilibrium path of ring analysis after the touchdown point.

The configuration of the most deformed generator of the tube (top generator) for different levels of material nonlinearity is presented in Fig. 18, showing the corresponding gap. The gap values are also summarized in Table 1. The gap size values are higher when the hyperelastic material model is used, which is attributed to the “hardening” behavior of the hyperelastic material model at higher strains (see Fig. 3a).

Furthermore, the level of material nonlinearity influences the length of the propagation profile, as shown in Fig. 18. The results show that increasing material nonlinearity in terms of exponent *n*, the propagation profile length $L_{p}$ decreases and the longitudinal profile of the generator becomes steeper. The value of $L_{p}$ is equal to $10.9D_{{{\text{o}}}}$, $9.3D_{{{\text{o}}}}$ and $7.3D_{{{\text{o}}}}$ for *n* equal to 2.4, 2.6 and 2.8, respectively. For $n=2$ such a length cannot be calculated, due to the short range of the unstable post-buckling regime. Additional numerical analysis performed with exponent value (*n*) equal to 5, results in further reduction of $L_{p}$ equal to $5.1D_{{{\text{o}}}}$ which is comparable to the one observed in steel pipes under external pressure (Kyriakides and Lee 2020).

### Influence of the diameter-to-thickness ratio (*D*/*t*)

The effect of *D*/*t* ratio on the post-buckling mechanism and the values of collapse pressure and propagation pressure are examined using ring and 3*D* tube models. Tubes with *D*/*t* ratio equal to 9, 12 and 30 are considered with nonlinear elastic material behavior.

The equilibrium paths in Fig. 19, obtained from ring models with $n=2.4$, show the variation of external pressure *P* with the change of area enclosed by the ring $\Delta A$. The case of $D/t=9$ is a thick-walled tube, which is representative of a physiological conduit (Kozlovsky et al. 2014). For all cases, the response exhibits a pressure maximum (collapse pressure), while beyond the maximum the pressure drops down to a minimum followed by a positive slope branch. For all cases, the minimum pressure ($\bigtriangleup$) occurs before contact of inner wall takes place, and is followed by an increase of pressure due to “stiffening”, as shown in Fig. 4. The shape of all responses enables the Maxwell line construction as described in Sect. 3.1. Using this method, the normalized value of propagation pressure is calculated equal to 0.615, 0.685, 0.809 and 0.886 for *D*/*t* value equal to 9, 12, 20 and 30, respectively. The actual level of propagation pressure in kPa for every case is summarized in Table 2. One may observe that the actual level of propagation pressure $P_{{{\text{p}}}}$ decreases with increase of the *D*/*t* value, whereas the normalized value of $P_{{{\text{p}}}}$ increases in terms of the *D*/*t* ratio. Figure 19 also presents the equilibrium paths using Marlow hyperelastic model, while the values of collapse pressure and propagation pressure are summarized in Table 2. The results show very good agreement between the two material models. Some differences between the two material models are obtained for the cases of *D*/*t* equal to 9 and 12, which may be attributed to the higher hoop stresses developed as the ring wall thickness is increased.

Figure 20 shows the variation of external pressure with respect to the change of the enclosed volume, obtained with the 3*D* model and $n=2.4$, confirming the conclusions drawn for the ring models as shown in Fig. 19. Furthermore, very good comparison between the two material models is observed. All diagrams develop a steady-state plateau corresponding to the value of propagation pressure. Table 3 summarizes the results of propagation pressure for every case, and the collapse pressure values are also included for completeness. One may notice that the propagation pressure values obtained from a ring model with the Maxwell line construction (Table 2) and a 3*D* tube model (Table 3) are very close, demonstrating the capability of the plane strain ring analysis to provide reliable estimations of the propagation pressure in a cost effective manner. The Maxwell line in ring model analysis intersects the equilibrium paths before the touchdown point for the cases with *D*/*t* equal to 12, 20 and 30, and therefore a residual gap exists in the propagation profile for each case. However, the Maxwell line in the ring model analysis with $D/t=9$ intersects the equilibrium path after the touchdown point, and thus contact is established during buckling propagation, as verified by the results of the 3*D* model.

The effect of material nonlinearity on the response and the post-buckling characteristics of rings with $D/t=12$ and $D/t=30$ cases is examined, following a similar analysis to the one presented in Sect. 5.1 for $D/t=20$. Figure 21 shows the variation of external pressure in terms of area change for increasing nonlinearity, obtained from ring models with *D*/*t* equal to 20. Similar to the $D/t=20$ case, increasing material nonlinearity (i.e., increasing the value of exponent *n*) results in more pronounced post-buckling instability, and this observation is consistent with the results from both material models. The corresponding equilibrium paths for the 3*D* tube models are shown in Fig. 22 in terms of the variation of external pressure with enclosed volume change. Increasing the nonlinearity results in a reduction of collapse pressure followed by a more pronounced post-buckling path. Consistent results are obtained for the tube with $D/t=30$, as shown in Figs. 23 and 24 for the ring and 3*D* tube models, respectively; the equilibrium paths follow the trends observed for the tubes with $D/t=12$ and 20, indicating that the effect of material nonlinearity on tube response under external pressure is rather independent of the *D*/*t* value from a qualitative point of view. Furthermore, Figs. 21 and 23 include the tube law expression of eq. (18), proposed by Kozlovsky et al. (2014), showing very good agreement with the linear elastic case ($n=1.0$) of each *D*/*t*. Tables 4 and 5 summarize the collapse pressure and propagation pressure values for the ring models and the 3*D* tube models, respectively, for the $D/t=12$ and $D/t=30$ cases with increasing nonlinearity and for the two material models.

### Effect of axial pre-strain

Axial pre-strain may be present in biological tubes and conduits, and influence their collapse and propagation response. This effect is investigated for tubes with $D/t=20$ and $n=2.4$, using the models of Sect. 2. Prior to external pressurization, in both ring and 3*D* models, the tube is initially elongated with an axial strain up to $3 \%$, imposing an axial displacement on the degrees of freedom of the end cross section ($z=L$) so that the desired level of pre-strain is reached. Subsequently, keeping the axial displacement constant, external pressure is applied.

Figure 25 presents the variation of external pressure with the area change and volume change, obtained from the ring and 3*D* model, respectively, for increasing levels of axial pre-strain. The results show that in both models the collapse pressure and the propagation pressure reduce with increasing the pre-strain, while the unstable part of the equilibrium path also reduces. Specifically, the normalized collapse pressure of the tube is 0.921 (0.937 kPa), 0.863 (0.878 kPa), 0.789 (0.803 kPa) and 0.728 (0.741 kPa) for 0%, 1.0%, 2.0% and 3.0%, respectively, while the normalized propagation pressure of the tube is 0.811 (0.824 kPa), 0.797 (0.811 kPa) and 0.767 (0.780 kPa) for 0%, 1.0% and 2.0%, respectively.

At 3.0% pre-strain, a slight pressure decrease occurs beyond a maximum pressure, followed by a stable branch of the equilibrium path. In the ring model, the Maxwell line construction predicts a normalized propagation pressure value equal to 0.723 (0.736 kPa). However, a steady-state buckle propagation is not achieved for the 3*D* tube model, and this is attributed to the very short (practically non-detectable) unstable part of the $P-\Delta A$ diagram.

The collapse pressure and propagation pressure values obtained for the tube and ring model are summarized in Table 6. Figure 26 presents the configuration of the most deformed generator of the tube model for different levels of axial pre-strain. The results show that the residual gap (*g*) between the upper and lower side of the inner surface of the tube and the length of the propagation profile ($L_{p}$) increase with increasing axial pre-strain level. Specifically, the propagation length reaches a value of $33.0D_{{{\text{o}}}}$ for pre-strain $2.0 \%$, and the corresponding residual gap is $34 \%$ of the tube radius. At 3.0% pre-strain, the propagation length and the residual gap could not been calculated, because steady-state buckle propagation may not be achieved, in the tube under consideration as noted above.

### Comparison with tubes made of inelastic material behavior

It is instructive to compare the response of elastic tubes with the response of tubes made of materials that exhibit irreversible (inelastic) deformation. This comparison is motivated primarily by the very good predictions of propagation pressure with the Maxwell line construction, in comparison with 3*D* model predictions, for all elastic tubes considered in this study (Section 3, and subsections 5.1, 5.2, 5.3). This good prediction in elastic tubes is in contrast with the rather poor predictions of propagation pressure obtained from Maxwell line construction in metal tubes.

Tubes with *D*/*t* equal to 20, and initial ovality equal to 0.2% are considered, with two types of inelastic material behavior. The first type is a soft material that follows a stress–strain curve similar to the one in Fig. 3 for loading, but exhibits purely inelastic behavior with unloading. This behavior is modeled using $J_{2}$ (von Mises) flow theory of plasticity, and due to the “exponential” shape of the curve, a very low yield stress ($\sigma _{y}=4$ kPa) is assumed. This material will be referred to as “inelastic” and its influence on tube response is examined in paragraph 5.4.1. The second type refers to actual steel pipe material, widely used in subsea pipelines (*X*65 steel grade). It is also modeled with $J_{2}$ flow theory of plasticity, it will be referred to as “metal material” and its effect is examined in paragraph 5.4.2.

#### Tubes that exhibit “inelastic” deformation

Figure 27 shows the variation of external pressure, with respect to the change of volume enclosed by the tube, obtained with the elastic and the inelastic material. In both material cases, the collapse pressure values are similar, while the propagation pressure presents some difference (0.808 versus 0.851). The results show that the deformed configuration of the most deformed generator along the tube model is similar for the two material models, and the propagation length $L_{p}$ of the top generator of the tube is also similar ($10.9D_{{{\text{o}}}}$). However, a certain difference is observed in the residual gap (*g*).

The propagation pressure of the 3*D* tube model with inelastic material is 0.854, and the corresponding value predicted from the ring model with the Maxwell line method is also very close 0.849. In this context, additional analyses are performed with the 3*D* tube model and the ring model, considering inelastic materials which have loading branches with different exponent values *n*. The normalized value of propagation pressure obtained from the ring model with *n* equal to 2.4, 2.6, 2.8, 3.5 and 5.0 is 0.849, 0.750, 0.652, 0.413 and 0.217, respectively. The corresponding values of $P_{{{\text{p}}}}$ obtained from the 3*D* tube model are 0.854, 0.755, 0.663, 0.439 and 0.254. The results show that the ring model underpredicts the propagation pressure by 0.6%, 0.8%, 1.7%, 6.0% and 14.8% with increasing value of the exponent *n*. This indicates that the accuracy of the Maxwell line construction in predicting the propagation pressure may depend on the amount of inelastic deformation that occurs during propagation. Additionally, the length of the propagation profile $L_{p}$ reduces significantly from $10.9D_{{{\text{o}}}}$ for $n=2.4$ to $5.6D_{{{\text{o}}}}$ for $n=5.0$. The reduction of the propagation length with increasing the value of exponent *n* is also observed for the elastic material. The propagation pressure is also obtained for nonlinear elastic material with *n* equal to 3.5 and 5.0; the normalized value for the ring model is 0.385 and 0.200, and for the 3*D* tube model is 0.384 and 0.200 for $n=3.5$ and $n=5.0$, respectively.

#### Metal tubes

To investigate further the effect of inelastic material behavior on the propagation pressure, the material of the tube under consideration is assumed to have the properties of an *X*65 steel tube material, commonly used in deep sea pipelines. Young’s modulus is 207 GPa, Poisson’s ratio is 0.3, and its stress–strain curve is obtained from a laboratory physical test (Gavriilidis et al. 2024). In this curve, the stress at $0.5\%$ strain is equal to 503 MPa. The material curve is employed in the finite element models (ring and 3*D*), considering a $J_{2}$ (von Mises) flow plasticity model with isotropic hardening.

The Maxwell line construction for predicting the propagation pressure is based on the assumption that the tube is composed of a sequence of rings, which collapse progressively, from the passage of the traveling buckle, as described in Sects. 3.1 and 4. The present results show that Maxwell line prediction for elastic tubes compares very well with the results from 3*D* analysis. However, Maxwell line estimates of propagation pressure for metal pipes are not satisfactory. For the case of *X*65 steel tube with $D/t=20$, the Maxwell line calculation underpredicts the propagation pressure by $30.4\%$ (6.17 MPa instead of 8.87 MPa obtained by the 3*D* model) confirming observations from previous publications in metal tubes (e.g., Kyriakides and Lee, 2020; Karamanos, 2022). On the other hand, the Maxwell line seems to constitute a reliable tool for predicting the value of propagation pressure in elastic tubes.

To explain this striking difference between elastic and metal tubes, the strain energy expressed by eq. (17) which considers the tube as an assembly of collapsible rings is compared with the corresponding strain energy obtained by a 3*D* analysis. Considering steady-state conditions of buckling propagation in a 3*D* analysis model, the energy $E_{t}$ necessary to advance the buckle by distance *L* (e.g., from $z_{o}$ to $z_{o}+L$ in Fig. 13d) is given by the following equation:

20 $$\begin{aligned} E_{t}= P_{{{\text{p}}}} (\Delta V|_{z_{o}+L}-\Delta V|_{z_{o}}) \end{aligned}$$

or equivalently from Figure 13d:

21 $$\begin{aligned} E_{t}= P_{{{\text{p}}}} (\delta \Delta A)L \end{aligned}$$

where $\delta \Delta A$ is the change in cross-sectional area of a ring from its initial to its final configuration during propagation. Thus, the energy required for the advancement of the buckle per unit length is

22 $$\begin{aligned} \hat{U}_{t}=E_{t}/L= P_{{{\text{p}}}} (\delta \Delta A) \end{aligned}$$

Using expressions (7) and (22), the values of $\hat{U}_{r}$ and $\hat{U}_{t}$ are calculated for an elastic tube made of nonlinear material with $n=2.4$ and for a metal tube with the *X*65 steel material. Both tubes have $D/t=20$. The values of $\hat{U}_{r}$ and $\hat{U}_{t}$, are shown in Table 7 normalized by the energy-like quantity $\hat{U}=Et^3/D_{{{\text{o}}}}$, and are very close for the tube made by elastic or by inelastic material (described in Sect. 5.4.1). On the other hand, the ring strain energy $\hat{U}_{r}$ of the metal tube is $36.5 \%$ lower than the energy per unit length of a traveling buckle $\hat{U}_{t}$, as shown in Table 7, and this is attributed to the longitudinal strain energy, not accounted for in the ring model. Comparing the deformed tube configurations in Figs. 1b and 9b, it is very clear that the generator profile of the elastic tube is significantly smoother than the generator of the metal tube. The corresponding values of the propagation length $L_{p}$ are equal to $10.9D_{{{\text{o}}}}$ and $5.4D_{{{\text{o}}}}$ for the elastic and the metal tube, respectively. From elementary mechanics, the shorter the length, the larger the longitudinal strain energy. Therefore, the longitudinal energy constitutes a substantial amount of total energy in metal tubes, whereas it is rather small compared with ring deformation energy in elastic tubes.

## Conclusions

The present study investigates the development of propagating instabilities in long collapsible tubes made of biological elastic material, subjected to uniform external pressure using two-dimensional (ring) and three-dimensional (3*D*) finite element models, and two constitutive models, which account for nonlinear elastic material behavior. Tubes with *D*/*t* values in the range of 9 to 30 are analyzed under quasi-static conditions, and the main findings of the study are summarized as follows.

The main conclusion is that propagating instabilities, also called “propagating buckles”, may occur in long tubes made of nonlinear elastic material with small deviations (softening) from linear elastic behavior. It is verified that tubes made of linear or quasi-linear elastic material collapse uniformly and do not exhibit localization and propagation instabilities, also noticed in previous publications. However, the numerical results show that there exists a minimum level of softening in nonlinear elastic materials, which allows for collapse localization (referred to as “buckle”) and its subsequent propagation along the tube. The presence of a maximum pressure on the equilibrium diagram followed by an unstable part is the key feature for collapse localization and propagation to occur, and is significantly influenced by the amount of material softening. Furthermore, the “buckle” propagates under steady-state condition at a pressure level called “propagation pressure”, which may be significantly lower than the maximum (collapse) pressure.

Application of the Maxwell line construction method in ring analysis provides accurate predictions of the propagation pressure, compared with the corresponding predictions of the more rigorous 3*D* analysis. Considering different levels of material softening, it is shown that localized collapse and its subsequent propagation may occur with or without contact of the inner pipe wall, and that in the latter case the size of the gap between the upper and lower side of the inner surface increases when the amount of material softening increases. Furthermore, when the intersection point (C) of Maxwell line with the equilibrium path of ring analysis occurs before the touchdown point (T), the “buckle” propagates without contact. On the other hand, contact of the inner surface occurs during propagation when the intersection point (C) is after the touchdown point (T). Additionally, in a ring analysis the value of cross-sectional (ring) deformation at point (C) coincides with the final value of cross-sectional deformation in a long tube during steady-state buckle propagation, obtained from 3*D* analysis. The above observations indicate a strong correlation between the ring behavior and the 3*D* tube response.

The presence of pre-strain in the longitudinal direction of the tube up to $3\%$ strain reduces both the collapse pressure and the propagation pressure, and “smoothens” the propagation profile. Tubes made of material that exhibits irreversible (inelastic) deformation are also analyzed, and their results are compared with the results from nonlinear elastic tubes. It is shown that the presence of irreversible material deformation during buckle propagation influences the value of propagation pressure, and this influence is more pronounced when the tube material exhibits significant softening in its loading branch.

Finally, comparison of elastic tubes with metal (elastic–plastic) tubes demonstrates that in elastic tubes the longitudinal strain energy necessary for buckle translation is significantly smaller compared to the hoop strain (ring collapse) energy, a result that is not observed in metal tubes. This difference can be also verified by comparing the corresponding shapes of the propagation profiles, and is the main reason for the very good predictions of the Maxwell line construction method in nonlinear elastic tubes, as opposed to its rather poor predictions in metal tubes.
